# Study on the persistence of ciprofloxacin and sulfamethoxazole in simulated drinking water systems

**DOI:** 10.1186/s40068-025-00396-5

**Published:** 2025-04-26

**Authors:** Victoria Rilstone, Yves Filion, Pascale Champagne

**Affiliations:** 1 Beaty Water Research Centre, Department of Civil Engineering, Queen’s University, Union Street, Kingston, K7L 3Z6 Canada; 2 Department of Chemistry, Queen’s University, Bader Lane, Kingston, K7L 2S8 Canada

## Abstract

The antibiotics ciprofloxacin and sulfamethoxazole are well-known to be persistent in drinking water, as they have been detected at the highest concentration and frequency, respectively. These antibiotics persist despite their residence time, water treatment, and environmental conditions encountered in drinking water distribution systems. To better understand this phenomenon, the objectives of this study were to determine their degradation kinetics at a residual, sub-minimum inhibitory concentration while exposed to multi-species biofilms in polyvinyl chloride (PVC) pipe, as well as examine their effect on total cell count (TCC). The results revealed that both antibiotics continued to be detected after the experimental period of 12 days. Ciprofloxacin concentrations decreased by 31.1% (± 3.9%) and 27.4% (± 7.7%) during exposure to the biofilm and PVC-only control respectively, whereas sulfamethoxazole concentrations decreased by 87.2% (± 15.8%) and 3.6% (± 8.6%) during exposure to the biofilm and PVC-only control, respectively. Biofilm TCC increased significantly when exposed to ciprofloxacin (*p*-value < 0.005), but showed no significant change when exposed to sulfamethoxazole (*p*-value > 0.05), which suggested that ciprofloxacin enhanced biofilm formation. These results address the gap in antibiotic persistence research by tracing their concentrations, elucidating the mechanisms of sorption and degradation, and discussing their relative implications. As antibiotics continue to persist in drinking water, their interaction with biofilms may contribute to the long-term selection of antibiotic-resistant bacteria, posing potential risks to drinking water safety and public health.

**Supplementary Information:**

The online version contains supplementary material available at 10.1186/s40068-025-00396-5.

## Introduction

Over the last two decades, the presence of antibiotics in water systems has been reported with increasing frequency in the research literature (Rilstone et al. 2021; Maghsodian et al. 2022). This accumulation in water systems can be attributed to several key causes such as increased global usage, environmental discharges, landfill leachates, and inadequate treatment. Between 2000 and 2015, antibiotic usage has increased globally by 65%, and it is projected to further increase to 200% by 2030 (i.e., from 21.1 to 34.8 billion to over 42 billion defined daily doses) (Klein et al. 2018). In addition, antibiotics can enter the water system through multiple sources, including direct disposal and/or excretion with partial metabolism into the sanitary sewer system, agricultural runoff, sorption/desorption from source water sediment, and industrial discharges (Manzetti and Ghisi 2014; Franklin et al. 2022). Once the antibiotics reach the wastewater treatment plant (WWTP), conventional treatment cannot achieve complete antibiotic removal (Polianciuc et al. 2020). As a result, advanced treatment technologies (e.g., adsorption, advanced oxidation processes, ozonation) are actively being developed and considered to address this issue (Phoon et al. 2020; Li et al. 2023a; Wang et al. 2024). However, their implementation remains limited as their effectiveness continues to be optimized, while their economic and scheduled feasibility will take several years to come to fruition (Olson 2023). Whether or not advanced treatment is applied, certain antibiotics may only be reduced to a lower residual level and/or continue to persist in drinking water (Rilstone et al. 2021; de Ilurdoz et al. 2022). The most notable of those antibiotics are ciprofloxacin and sulfamethoxazole, which have been found at the highest concentration and frequency in drinking water, respectively (Mahmood et al. 2019; Ye et al. 2007). Specifically, ciprofloxacin is a broad-spectrum semisynthetic fluoroquinolone that has been found at concentrations up to 0.492 μg L^−1^, whereas sulfamethoxazole is a sulfonamide that has been found at concentrations of up to 0.113 μg L^−1^ in drinking water (Mahmood et al. 2019; Ye et al. 2007).

To date, research on antibiotic persistence has primarily focused on two key areas: advanced treatment removal efficiencies and antibiotic monitoring in water systems. However, both areas have limitations, making it difficult to draw conclusions regarding the long-term effects of antibiotics being detected in a drinking water distribution system (DWDS). Specifically, when advanced treatment studies have reported the removal efficiencies of antibiotics from source water, they have been limited in detecting their reaction products and could not detect transition states and intermediates (Liu et al. 2022; Nie et al. 2022; Zhu et al. 2023; Zhang et al. 2024; Li et al. 2023b). When the degradation kinetics of the antibiotics are plotted, the time scale is often limited from minutes to hours (Karim et al. 2020; Guo et al. 2015; Feng et al. 2018), raising uncertainties regarding their long-term persistence. At the same time, these studies focused their treatment methods on antibiotics in the mg L^−1^ concentration range, which is not necessarily validation that the same removal efficiencies would be expected at residual levels (i.e., ng L^−1^ to μg L^−1^) (Escher et al. 2020). Conversely, there are antibiotic monitoring studies that are performed in the field and therefore examine antibiotics at realistic residual concentrations (Hanna et al. 2020; Bhagat et al. 2020). However, their limitations include their scope and confounding variables. For the scope of research, studies that focused on antibiotic monitoring were limited in their number of samples, as well as the length (i.e., months, years), time (i.e., season, sampling frequency), and location (i.e., geographic region, type of water system) of study (Watkinson et al. 2009; Wang et al. 2019; Ngigi et al. 2020). Additionally, when studies are conducted in the field, the antibiotics are moving in an open system and are subject to inconsistent dilutions and uncontrollable and often unknown environmental factors. With such limitations, it is difficult to accurately trace and standardize antibiotic persistence. Altogether, research has yet to investigate the long-term behaviour of antibiotics at residual concentrations, while in a controlled environment.

With the increasing rate of antibiotic usage, ongoing detections in drinking water, and a limited understanding of their long-term persistence, there is a growing concern regarding human exposure, and by extension, their ability to promote antibiotic resistance. At residual levels, antibiotic concentrations may behave differently depending on the water quality and microbiological parameters of a drinking water system. As antibiotics travel through water systems, they are inevitably exposed to bacteria and biofilms that grow along pipe walls. This factor is often overlooked by researchers and practitioners. Antibiotics may also be present at a sub-minimum inhibitory concentration (sub-MIC), which can promote antibiotic resistance. In 2019, it was found that antibiotic resistance was directly responsible for 1.27 million global deaths (World Health Organization 2023). When bacteria and biofilms are exposed to antibiotics for a prolonged duration, the probability of resistance promotion increases (Mo et al. 2023), further emphasizing the importance of examining their long-term behaviour in drinking water.

The current work extends previous work on antibiotic degradation (Karim et al. 2020; Guo et al. 2015; Feng et al. 2018) by examining the persistence of two common antibiotics over an extended period of time. It also builds on previous field work in antibiotic persistence (Watkinson et al. 2009; Wang et al. 2019; Ngigi et al. 2020) by examining the persistence of antibiotics in a fully controlled environment—something that is not possible in field studies. The aim of the paper is to create a semi-closed drinking water environment, consisting of representative water quality conditions to examine the persistence and behaviour of the antibiotics that have been most documented in drinking water systems (i.e., ciprofloxacin and sulfamethoxazole). The specific objectives of this study are to: (1) determine the degradation kinetics of ciprofloxacin and sulfamethoxazole over 12 days at a residual, sub-MIC concentration when exposed to multi-species biofilm formed on polyvinyl chloride (PVC) pipe, and; (2) investigate their effects on total cell count (TCC), which was used as an indicator of biofilm growth. The results of this research will better our understanding of antibiotic persistence to create better risk assessments, provide a scientific basis for the development of environmental policies and regulations, and further incentivise advanced water treatment research and implementation.

## Methods and materials

### Reagents and materials

For this research, ciprofloxacin (purity > 98%) and sulfamethoxazole (purity > 98%) were purchased from ThermoFisher Scientific (MA, USA) and Millipore Sigma (MO, USA), respectively. Stock solutions for ciprofloxacin and sulfamethoxazole were separately prepared by weighing and dissolving requisite amounts of each compound in deionized water (DI water) under magnetic stirring at 1600 rpm for 72 h to yield concentrations below 10 mg L^−1^. The ciprofloxacin stock solution was prepared in the dark at room temperature (23 °C), whereas the stock solution for sulfamethoxazole was prepared in the dark at 4 °C. Each stock solution was subsequently filtered using a 0.2 μm pore size Nylon syringe filter to remove any solid form from the liquid phase and stored in the dark at 4 °C for a maximum of 14 days. The physico-chemical properties of the two antibiotics are presented in Table 1.

**Table 1:** Physico-chemical properties of ciprofloxacin and sulfamethoxazole

Antibiotic	Chemical structure	Molecular formula	Molecular weight	Unmetabolized fraction (%)		pK_a_	logK_ow_	References
Ciprofloxacin		C_17_H_18_FN_3_O_3_	331.34	50–83.7		6.09, 8.74	0.28	Mompelat et al. (2009)
Sulfamethoxazole		C_10_H_11_N_3_O_3_S	253.28	15–30		1.6, 5.7	0.89	Mompelat et al. (2009)

pK_a_ = the negative log of the acid dissociation constant, logK_ow_ = the logarithm of the octanol/water partition coefficient

### Experimental conditions

**Fig. 1:**
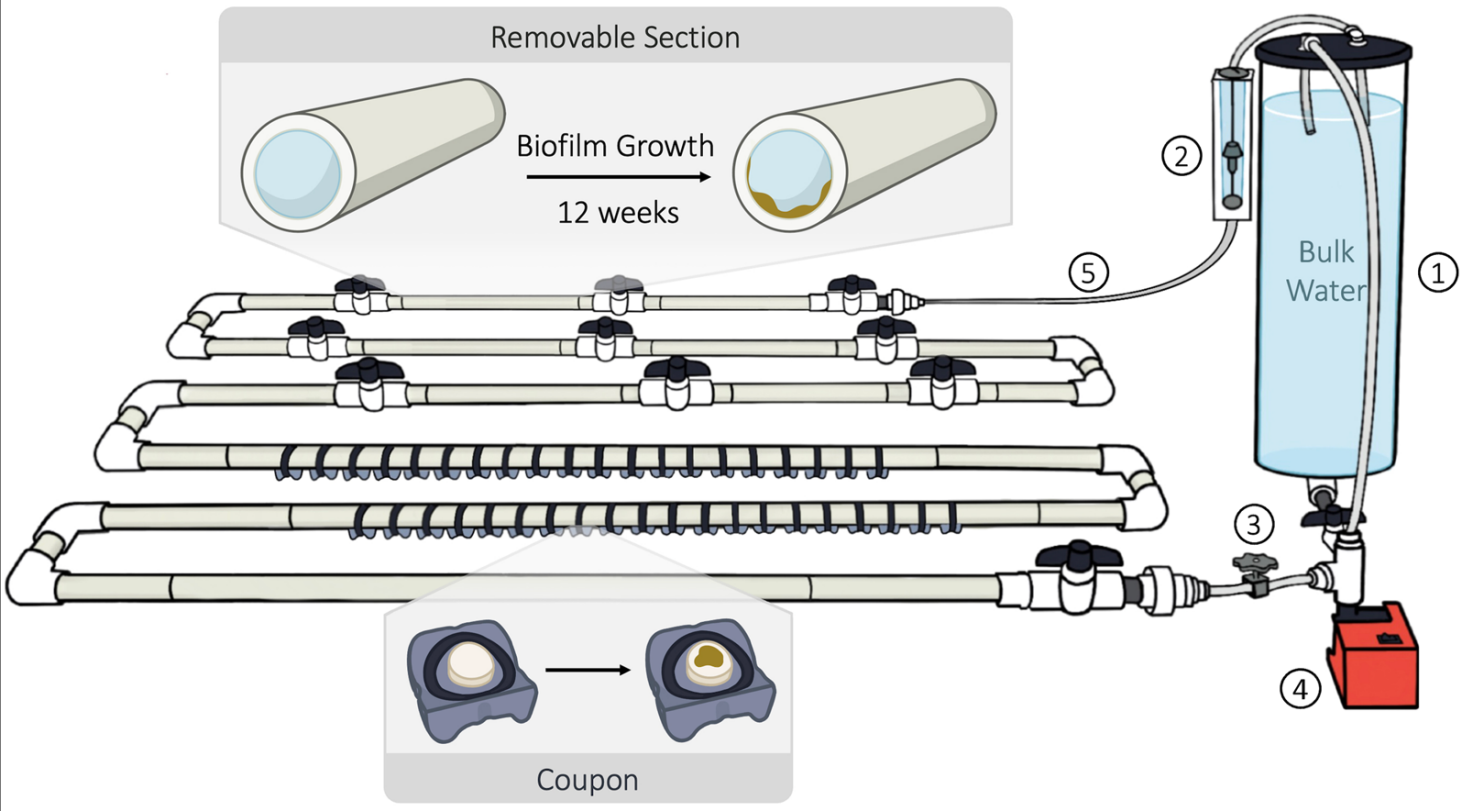
Schematic diagram of one bench-scale water distribution reactor (BWDR). One BWDR consists of a 4 ¼-inch (10.80 cm) ID PVC reservoir and 9 m of looping ¾-inch (1.91 cm) ID PVC pipe, circulating a total of 7 L of bulk water. Along the pipes are removable sections as well as coupons with a PVC sample disk that fit into holes drilled along the invert of the pipe. Each BWDR has (1) a 5 L PVC reservoir, (2) an adjustable flow rate meter, (3) a 3D-printed valve to control the flow rate, (4) a centrifugal pump (24 V DC), and (5) ¼-inch (0.64 cm) ID black vinyl tubing connecting the reservoir to the piping. BWDRs are run for 12 weeks to generate mature biofilm prior to their experimental period

A bench-scale water distribution reactor (BWDR) was designed and constructed to simulate and replicate premise plumbing hydraulics, water chemistry, and microbial conditions for researchers to better control the environmental parameters, as well as obtain samples through a non-intrusive sampling system. The BWDR was comprised of six 1.2 m long ¾-inch nominal size (nominal diameter, 0.824 inch ID, 1.05-inch OD) SCH40 PVC pipes, as well as a 7 L PVC reservoir (Fig. 1). For non-intrusive sampling, the pipe segments were divided into five removable segments separated by valves, as well as segments containing 40 coupons located along the pipe invert.

**Fig. 2:**
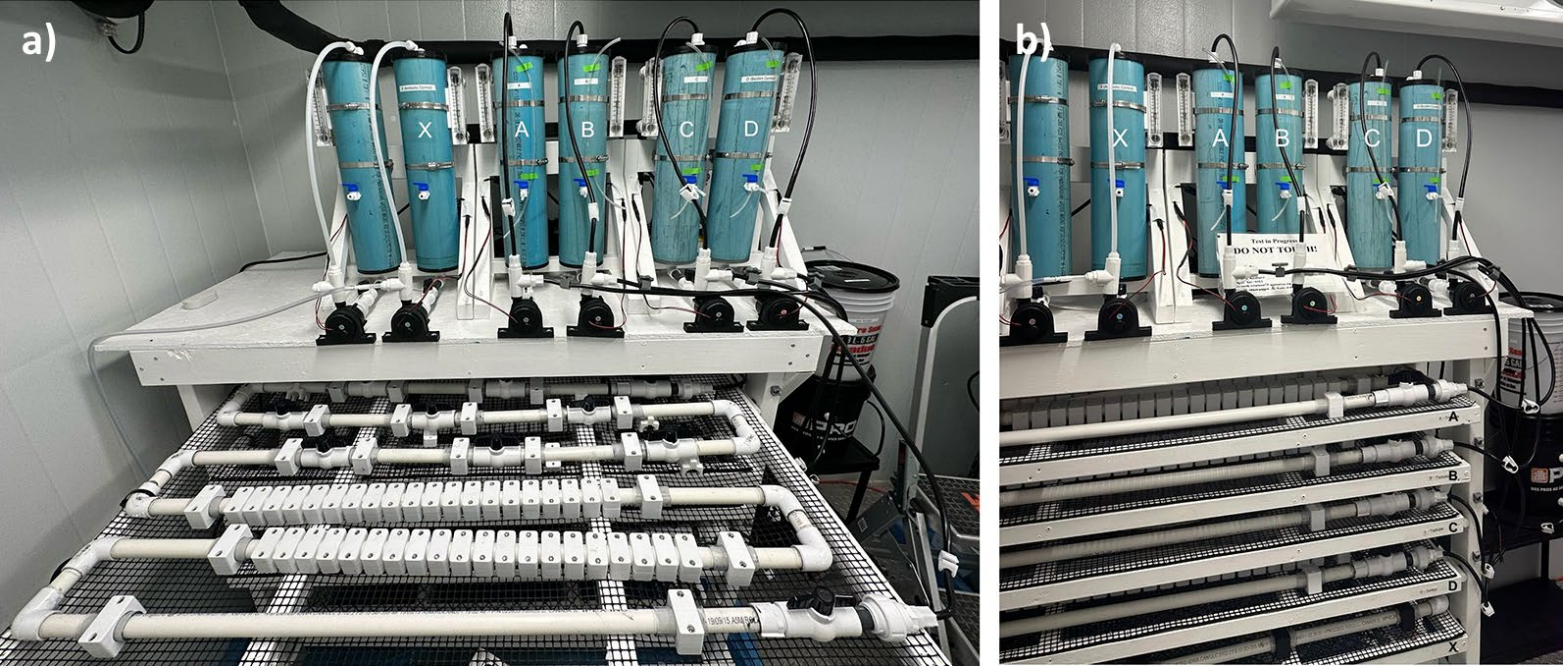
The experimental set-up of the 5 bench-scale water distribution reactors (BWDRs) on a mounted shelving system. The reservoirs that are part of each BWDR from right to left, excluding the first blank reservoir are: X (antibiotic control, referred to as PVC-only control), A, B, C and D (biofilm control)

Five BWDRs were operated synchronously for each antibiotic experiment, where three BWDRs functioned as experimental replicates (A, B, and C) and two as controls (i.e., biofilm with no antibiotic control (D) and antibiotic with no biofilm control (X), referred to as PVC-only control) (Fig. 2). The bulk water that was used in the BWDRs A, B, C, and D was taken directly from the output of two granular activated carbon (GAC) columns that were connected in series, each containing 0.86 kg of coconut shell high-activated carbon (Clack Corps., USA), that were fed a continuous supply of water from a tap on-site in Kingston, Ontario, Canada. The average water quality conditions taken from the GAC output are presented in Table 2. The first GAC column served to filter and remove the free chlorine from the tap water, while the second GAC column served to accumulate native microorganisms to re-saturate the output water with planktonic bacteria. The planktonic bacteria present in the output of the GAC were representative of those typically found in drinking water. The output water from the GAC was used to grow independent biofilms in each of the BWDRs (i.e., A, B, C, and D). This output GAC water, hereon referred to as bulk water once transferred into the designated BWDRs, was kept at room temperature, between 20 and 22 °C, and ran at a turbulent flow rate of 3 L min^−1^ (Reynolds number = 3000) for 12 weeks. The bulk water was circulated for 2 week periods and then replaced at the end of each 2 week period to allow sufficient time for bacterial attachment to pipe walls. Replacing the water every 2 weeks made it possible to achieve bulk water quality conditions that were more representative of those found in actual drinking water systems, as it would reset the water quality parameters to those in Table 2. For this reason, the BWDRs were considered to be continuous, semi-closed-loop systems. Nutrient broth (No. 3, NutriSelect^®^ Plus, Millipore Sigma, MO, USA) was administered at the start of each week at a concentration of approximately 0.24 g L^−1^ to expedite bacterial growth, replication, and subsequent biofilm formation on the pipe wall. During the entirety of the experimental period, the water quality parameters were continuously measured and monitored. A complete table of the water quality parameters and their associated monitoring methods can be found in Table S1 and Text S1 of the Supplementary Material, respectively.

**Table 2:** Average water quality of effluent from the granular activated carbon (GAC) output that is connected to the on-site drinking water tap

Temperature (°C)	pH	Turbidity (NTU)	UV absorb at 600 nm (A)	TOC (mg L^−1^)	TN (mg L^−1^)	Free Cl (mg L^−1^)	ATP (pg mL^−1^)	TCC (cell mL^−1^)
20.8	7.47	0.19	0	1.98	0.47	0.02	19.29	2130

Each value is the mean of n = 15 sample points taken every 2 weeks during the biofilm growth phase when the water was replaced

### Antibiotic administration

Once the biofilm was stabilized and reached its presumed maturity, after the 12 week growth period, each antibiotic was administered at a concentration of 10 μg L^−1^ by pipetting the appropriate volume from each stock solution. For each antibiotic experiment, the antibiotic was administered to BWDRs A, B, C, and X, where A, B, and C contained mature biofilm while X was the PVC-only control, only containing DI water and no biofilm. BWDR D was run in parallel but maintained as the biofilm control with no antibiotic added. Similarly, in an independent set of experiments, the same BWDRs were sterilized and used for the sulfamethoxazole experiment. Each stock solution was administered after a maximum of 1 day after it was prepared. Stability tests were conducted during the duration of both experiments. The stability tests confirmed that the antibiotics remained unaltered by the temperature and water quality parameters.

### Antibiotic concentration justification

A concentration of 10 μg L^−1^ was selected for this experiment for four reasons: (1) an independent literature review was conducted and found that the most common species of DWDS biofilms had an inhibitory concentration above 10 μg L^−1^, which suggests that this concentration would produce similar behaviours and effects to those found at documented residual levels, (2) the aforementioned maximum reported concentrations for ciprofloxacin and sulfamethoxazole were 0.492 μg L^−1^ and 0.113 μg L^−1^ respectively (Mahmood et al. 2019; Ye et al. 2007), which were considered to be comparable to the selected concentration, (3) currently, there are no nationally- or internationally-regulated limits for antibiotics in drinking water (Schwartz et al. 2021; Wang et al. 2023), and (4) to minimize error. For instance, concentrations lower than 1 μg L^−1^ would require sample clean-up methods (such as solid-phase extraction (SPE)). However, when SPE was tested, there was a low recoverability and extractability, making the results less reproducible. Therefore, the selected concentration of 10 μg L^−1^ limits error by maximizing the specific observable changes by selecting a starting value that can produce reproducible subsequent detectable values.

### Antibiotic concentration sample collection

Immediately after the antibiotic was administered to each respective BWDR (i.e., A, B, C, and X), the bulk water was circulated for approximately 10 min to allow the antibiotic to homogenously disperse for accurate concentration readings. Bulk water samples were then taken at 10 min, 1 day, 6 days, and 12 days after the antibiotic was introduced into the BWDR. To collect the bulk water samples, the pump for each BWDR was briefly turned off to collect n = 3 biological replicates from each individual BWDR at different reservoir heights at each time. A pipette was used to collect 5 mL of bulk sample water for each time point and dispensed into three separate 15 mL sterile falcon tubes. Each sample was protected from direct light and centrifuged at 3800 rpm for 10 min to create a pellet to separate the antibiotic present in the supernatant from the biological material. The supernatant was then filtered through a 0.2 μm pore size Nylon syringe filter and stored in a 2 mL amber glass autosampler vial (9–425 screw-thread, white PTFE & red silicone Septa screw cap) in the dark at 4 °C for a maximum of 3 days.

### Analytical methods

The bulk water samples that contained each antibiotic were analyzed with a ThermoFisher Exploris 120 Orbitrap mass spectrometer connected to a Vanquish ultra-high-performance liquid chromatography (uHPLC) system, with the autosampler held at 10 °C to preserve sample stability. Analytes were separated using a 50 mm × 2.1 mm × 3.0 µm Zorbax C18 analytical column paired to a corresponding guard column. Mobile phases consisted of 0.1% formic acid in LC–MS grade water (A) and LC–MS grade acetonitrile (B). For the analysis of ciprofloxacin, a gradient elution profile was used, starting at 90:10 A: B for the first 30 s, before shifting to 10:90 A: B over the next 2.5 min (three minutes total), holding at 10:90 A: B for one minute, before re-equilibrating at starting conditions for three minutes. For the analysis of sulfamethoxazole, an isocratic elution profile was used of 70:30 A: B at a rate of 0.3 mL min^−1^ over a 7.5 min duration. A diverter valve was employed to send the first 30 s of elution to waste.

After separation, analytes were ionized using a heated electrospray ionization source with a static spray voltage, running a positive ion voltage of 2500 V with an Orbitrap resolution of 30,000. Gasses were run in static mode, with a sheath gas of 50 (AU), auxiliary gas of 10 (AU), and sweep gas of 1 (AU). The ion transfer tube temperature and vaporizer temperature were set to 325 °C and 350 °C, respectively. The MS Global settings were set to expect an LC peak width of 10 s, and mass calibration was performed using the RunStart Easy-IC ™ internal calibration system. Analyte peak areas were acquired in full scan mode, with extraction ion chromatograms used to calculate the peak area for quantification purposes. Analyte peak area was determined using XCalibur QuanBrowser, monitoring for m/z 332.1404 for ciprofloxacin (+) and 254.0594 for sulfamethoxazole (+). Single ion monitoring (SIM) was also used to the accuracy of the EIC peak areas, which were found to differ by less than 5%.

Analytes were quantified using an external calibration curve of 0.26 μg L^−1^ to 52 μg L^−1^ (R^2^ = 0.999) for ciprofloxacin and 0.42 μg L^−1^ to 84 μg L^−1^ (R^2^ = 0.999) for sulfamethoxazole. The calibration curves were based on a set of serial dilutions created by each respective stock solution. Quantification limits were set to the lowest concentration of each calibration curve.

### Biofilm collection, quantification, and identification

Coupon samples (surface area 0.78 cm^2^) with biofilm adhered to them were extracted before and during exposure to each antibiotic in n = 3 biological replicates for every time point of 0, 1, and 12 days. Each coupon was rinsed with DI water and swabbed with a sterile swab, which was then placed in a fixative solution of 100 μL of glutaraldehyde and 9.9 mL of DI water. To quantify the biofilm, the Bacterial Counting Kit for flow cytometry (Invitrogen, Carlsbad, CA USA) was used according to the manufacturer’s protocol and analyzed on a SH800 cell sorter (Sony, Japan) to obtain TCC data. The resulting data analysis was performed using FlowJo^™^ (BD Biosciences, NJ, USA).

To obtain sufficient biomass for the 16S rRNA sequencing to identify the biofilms’ bacteria, the removable pipe segments (surface area 151.29 cm^2^) were also extracted at each time point of 0, 1, and 12 days. Each pipe segment was rinsed in DI water via vertical submersion and swabbed with sterile swabs until complete removal of visible biofilm. Swabs were individually vortexed to form pellets that were used for DNA extraction. DNA was extracted with the Sox DNA Isolation Kit (Metagenom Bio Inc.) according to the supplier’s recommendation. The detailed 16S rRNA methods can be found in Text S2 of the Supplementary Material.

### Statistical analysis

*Antibiotic degradation kinetics and biofilm TCC analysis:* The Shapiro–Wilk test was used on all datasets to determine normality as all datasets had a sample size of less than 50. A dataset was considered to be the exact variables for a specific time point or rate of change. The mean of all individual experimental replicates was used to generate a proportional and consistent dataset for comparisons to the control. For the independent kinetic degradation and TCC datasets (e.g., [ABC] versus [X]), if the null hypothesis was accepted (*p*-value > 0.05), then the parametric Student t-test was used. If the null hypothesis was not accepted (*p*-value < 0.05), then the non-parametric Mann–Whitney U test was used. For the dependent kinetic degradation and TCC datasets (e.g., [ABC]_0.0_ vs. [ABC]_0.1_, where the subscript denotes the time point), if the null hypothesis was accepted (*p*-value > 0.05), then the parametric paired samples t-test was used. If the null hypothesis was not accepted (*p*-value < 0.05), then the non-parametric Wilcoxon signed-rank test was used. To observe overall dependent changes (e.g., [ABC]_0.0_ to [ABC]_12.0_), if the null hypothesis was accepted (*p*-value > 0.05), then the parametric repeated measures ANOVA was used, whereas if the null hypothesis was not accepted (*p*-value < 0.05), then the non-parametric Friedman test was used. All kinetic degradation data accepted the null hypothesis except for sulfamethoxazole’s overall change in dependent [ABC] measures. The TCC data had high positive skewness and heterogeneity in its variance and was therefore transformed using the base-10 log, resulting in all datasets accepting the null hypothesis. Base-10 log transformations were applied before determining rates of change to preserve signage for slope/rates of change. Data analyses were conducted using Jamovi version 2.4.12.

*Correlation coefficient analysis:* To determine the correlation coefficient of antibiotic kinetic degradation concentration to the biofilm TCC, the rates of change between time 0 to 1 day and 1 to 12 days were used. The datasets to compute the correlation coefficient were the respective rates of change for each of the individual experimental replicates (i.e., [A], [B], and [C]), providing n = 18 matched pairs. Given the non-linear nature of the plotted data and the presence of outliers, a Spearman Rank correlation coefficient was used to characterize the correlation between the change in antibiotic concentration and the change in biofilm TCCs. Unlike the Pearson correlation, which assumes a linear relationship and normally distributed data, Spearman’s correlation is rank-based and better suited for capturing monotonic relationships in non-parametric data. Data analyses were conducted using Jamovi version 2.4.12.

*Community composition comparison:* To standardize the data, the 16S rRNA genus data for both antibiotic experiments from days 0, 1, and 12 for [A], [B], [C], and [D] were separately converted to relative abundances based on the total number of genera in each individual sample, excluding unassigned. The relative abundances for each BWDR (e.g., [A_Day 0_], [A_Day 1_], and [A_Day 12_]) where n = 3 were combined to create an overall average relative abundance. Each relative abundance was then scaled from 0.0 to 1.0. The Bray–Curtis dissimilarity index was then calculated between each BWDR to determine the dissimilarity between and within BWDRs. In addition, the Jaccard similarity index was used to calculate the similarity between and within BWDRs based on presence-absence data by converting the abundance values into binary form.

It is important to note that the following significance values that will be reported for the antibiotic degradation kinetics and biofilm TCC results each had a small sample size of n = 3 for each time point in each condition and should be interpreted with a low level of confidence.

## Results

### Degradation kinetics of ciprofloxacin and sulfamethoxazole

**Fig. 3:**
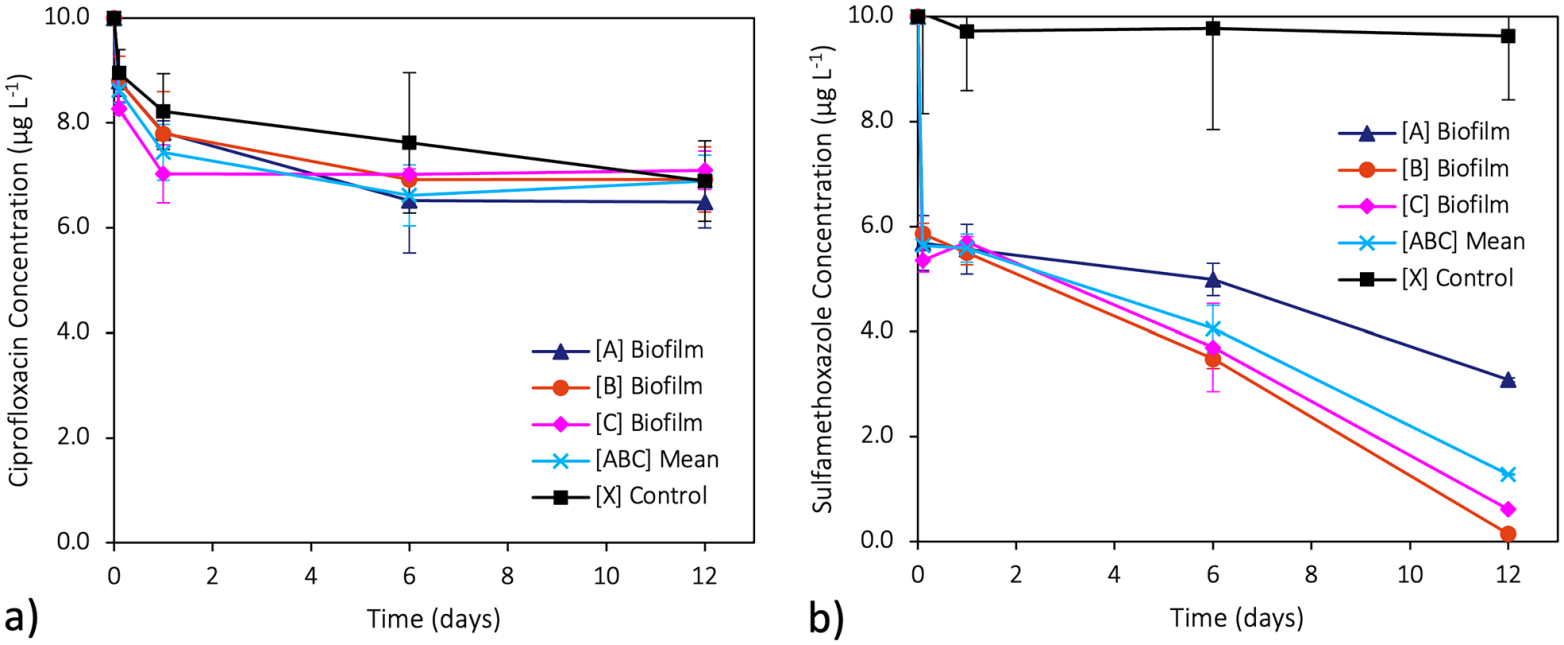
Kinetic degradation curves for **a**) ciprofloxacin and **b**) sulfamethoxazole when exposed to biofilm. [A], [B], and [C] are the concentrations of the antibiotic in individual experimental replicates containing biofilm. [ABC] is the mean of the antibiotic concentrations in the experimental replicates, and [X] is the concentration of the antibiotic with no biofilm present. Each point represents the mean (n = 3 independent samples), and the error bars indicate the standard deviation. The solid lines represent the rate of change

The kinetic degradations of ciprofloxacin and sulfamethoxazole are shown in Fig. 3, where [A], [B], and [C] are the concentrations of the antibiotic while exposed to biofilm. [ABC] is the mean concentration of the experimental replicates, and [X] is the control concentration of the antibiotic with no biofilm present. For each antibiotic, the subscripts CIP and SMX are used for ciprofloxacin and sulfamethoxazole, respectively. A complete table of the antibiotic concentration raw data and rates of change can be found in Tables S2 and S3 of the Supplementary Material, respectively.

In Fig. 3a, the kinetic degradation of ciprofloxacin is shown. Here, the overall concentration in both [ABC_CIP_] and [X_CIP_] significantly decreased after 12 days (*p*-values < 0.0001 and < 0.001 respectively). At the same time, there was no significant difference between [ABC_CIP_] and [X_CIP_] at each time point (*p*-value > 0.05). Between dependent sample time points in both [ABC_CIP_] and [X_CIP_], the rate of degradation of ciprofloxacin decreased over time. After 10 min, 1 day, and 6 days, the decrease in [ABC_CIP_] was statistically significant (*p*-values ≤ 0.01, < 0.05, and ≤ 0.01 respectively, where each *p*-value indicates the significance between each listed time point), whereas after 6 days, no statistically significant difference was noted ([ABC_CIP_] *p*-value > 0.05). Conversely, while there was a steady decrease between each of the dependent time points in [X_CIP_], its differences were not statistically significant (*p*-values > 0.05).

In Fig. 3b, the kinetic degradation of sulfamethoxazole is shown. It can be seen that the overall concentration of [ABC_SMX_] significantly decreased (*p*-value < 0.0001), while the concentration of [X_SMX_] did not significantly decrease (*p*-value > 0.05) after 12 days. At each time point of 10 min, 1 day, 6 days, and 12 days, the differences between [ABC_SMX_] and [X_SMX_] were significantly different (*p*-values < 0.05, < 0.005, < 0.01, and < 0.0001 respectively). Between the dependent sample time points in [ABC_SMX_], the rate of degradation of sulfamethoxazole exhibited a significant sharp initial decrease after 10 min (*p*-value < 0.001). It was briefly stable between 10 min to 1 day (*p*-value > 0.5), and then significantly and steadily declined after 1 day and 6 days (*p*-values < 0.05 and < 0.005 respectively). Conversely, [X_SMX_] did exhibit a decrease and the differences between each of its dependent time points were not significant (*p*-values > 0.5). A complete table of the significance test scores can be found in Table S3 of the Supplementary Material.

### TCC in biofilms exposed to ciprofloxacin and sulfamethoxazole

**Fig. 4:**
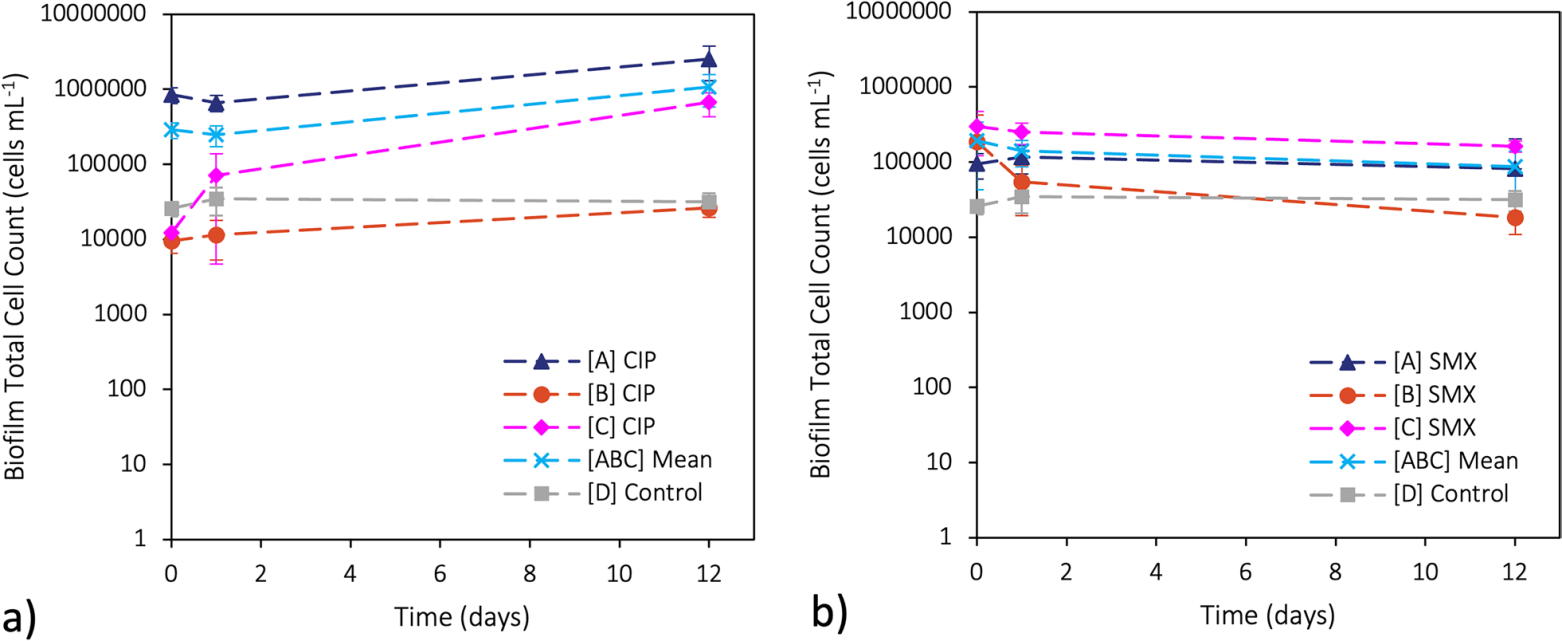
Biofilm growth curves with base-10 logged total cell count (TCC) when exposed to **a**) ciprofloxacin (CIP) and **b**) sulfamethoxazole (SMX). [A], [B], and [C] are the concentrations of biofilm TCCs in individual experimental replicates exposed to the antibiotic, [ABC] is the mean of the experimental replicates’ biofilm TCCs, and [D] is the concentration of the biofilm TCCs with no antibiotic present. Each point represents the mean (n = 3 independent samples), and the error bars indicate the standard deviation. The dashed lines represent the rate of change

The TCCs in the biofilms exposed to ciprofloxacin and sulfamethoxazole are shown in Fig. 4, where [A_TCC_], [B_TCC_], and [C_TCC_] are the concentrations of TCCs while exposed to the antibiotic, [ABC_TCC_] is the mean of the TCCs in three experimental replicates, and [D_TCC_] is the control concentration of TCCs with no antibiotic present. For each antibiotic experiment, the subscripts CIP and SMX were used for ciprofloxacin and sulfamethoxazole, respectively. Since the difference between the mean of the initial TCC concentration in the antibiotic experimental replicates was significantly different from the initial TCC concentration in the control (*p*-value < 0.005 for ciprofloxacin and < 0.01 for sulfamethoxazole), comparisons between their independent rates of change were made. The base-10 log-transformed values were used to examine the rates of change to standardize the distribution of the TCC data for more accurate comparison. To preserve randomness in calculating rates of change and their significance, triplicate samples were taken with noted sampling orders. This ensured the proper pairing of samples from different time points, maintaining randomness for accurate slope calculations. A complete table of the transformed TCC data and rates of change can be found in Tables S4 and S5 of the Supplementary Material respectively.

In Fig. 4a, the experimental biofilms were exposed to ciprofloxacin. The overall mean rate of change in [ABC_TCC-CIP_] from day 0 to day 12 compared to [D_TCC-CIP_] was significant (*p*-value < 0.05), where the overall rate of change of [ABC_TCC-CIP_] (0.05 cells mL^−1^ day^−1^) was greater than the overall mean rate of change of [D_TCC-CIP_] (0.01 cells mL^−1^ day^−1^). More specifically, the difference in the rate of change between [ABC_TCC-CIP_] compared to [D_TCC-CIP_] after 1 day was not initially significant (*p*-value > 0.05), where the mean slope of [ABC_TCC-CIP_] was -0.07 cells mL^−1^ day^−1^ and the mean slope of [D_TCC-CIP_] was 0.11 cells mL^−1^ day^−1^. However, between 1 and 12 days, this difference became significant (*p*-value < 0.005), where the mean slope of [ABC_TCC_] was 0.06 cells mL^−1^ day^−1^ and the mean slope of [D_TCC-CIP_] was -0.002 cells mL^−1^ day^−1^. Between dependent sample time points, the overall difference in TCCs within [ABC_TCC-CIP_] from day 0 to day 12 showed a significant increase (*p*-value < 0.005). After 1 day, the difference in TCCs [ABC_TCC-CIP_] was not significant (*p*-value > 0.05), whereas the difference in TCCs between 1 and 12 days was significantly different (*p*-value < 0.05). Conversely, the difference within [D_TCC-CIP_] was neither significant overall nor between individual time points.

In Fig. 4b, the experimental biofilms were exposed to sulfamethoxazole. The overall rate of change in [ABC_TCC-SMX_] from day 0 to day 12 compared to [D_TCC-SMX_] was not significant (*p*-value > 0.05), where the overall mean rates of change in [ABC_TCC-SMX_] and [D_TCC-SMX_] were -0.03 cells mL^−1^ day^−1^ and 0.01 cells mL^−1^ day^−1^, respectively. The difference in the rates of change between [ABC_TCC-SMX_] and [D_TCC-SMX_] was consistently not significant (*p*-value > 0.5) between all the time points, where the mean slopes after 1 day and between 1 and 12 days for [ABC_TCC-SMX_] were -0.06 cells mL^−1^ day^−1^ and -0.02 cells mL^−1^ day^−1^, respectively; and for [D_TCC-SMX_] were 0.11 cells mL^−1^ day^−1^ and -0.002 cells mL^−1^ day^−1^, respectively. Between dependent sample time points, the overall and time-specific differences within both [ABC_TCC-SMX_] and [D_TCC-SMX_] were all not significant (*p*-value > 0.05). A complete table of the significance test scores can be found in Table S6 of the Supplementary Material.

### Correlation between antibiotic kinetic degradation and TCC

The relationship between the rate of kinetic degradation for each antibiotic and their respective changes in TCCs between each time point was negative. However, neither antibiotic had a significant correlation between its kinetic rate of degradation and TCC. Specifically, the correlation between the kinetic degradation rate of ciprofloxacin and TCC was not statistically significant (correlation of -0.117, *p*-value > 0.05). The correlation between the kinetic degradation rate of sulfamethoxazole and TCC was not statistically significant (correlation of -0.169, *p*-value > 0.05).

### Biofilm community composition

**Fig. 5:**
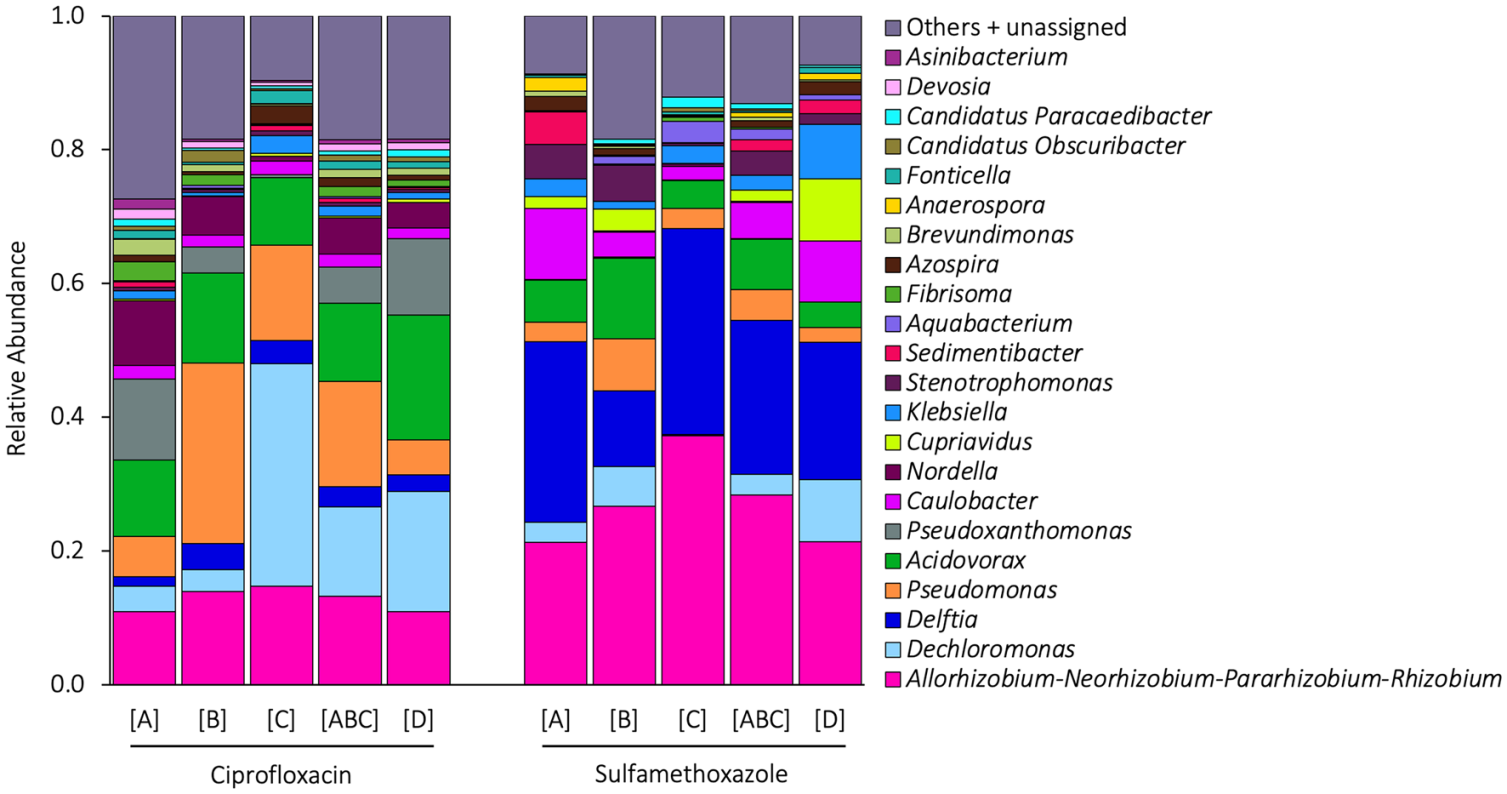
The relative abundance of the biofilm community composition at the genus level for the ciprofloxacin and sulfamethoxazole experiment. [A], [B], and [C] are the compositions of the biofilms in the individual experimental replicates that were exposed to the antibiotic, [ABC] is the mean composition of the individual replicates’ biofilms, and [D] is the composition of the control biofilm that was not exposed to an antibiotic within the same experimental period. Each biofilm composition presented consists of the average (n = 3 samples) genera at day 0, day 1, and day 12 for each bench-scale water distribution reactor (BWDR). Genera with a relative abundance of 0.01 or less was placed in “Others + unassigned”

The biofilm community composition for each antibiotic experiment is indicated in Fig. 5. The average dominant genera (relative abundance of ≥ 0.05 in at least one antibiotic experiment) for the ciprofloxacin and sulfamethoxazole experiments were *Allorhizobium-Neorhizobium-Pararhizobium-Rhizobium* (12.6% and 26.7%), *Dechloromonas* (14.6% and 4.6%), *Delftia* (2.8% and 22.4%), *Pseudomonas* (13.1% and 6.6%), *Pseudoxanthomonas* (7.0 and 0.1%), *Caulobacter* (1.8% and 6.4%), and *Nordella* (5.0% and 0.1%), respectively. A total of 106 genera were identified throughout both experiments. A complete table of the genus compositions can be found in Tables S7 and S8 of the Supplementary Material.

**Fig. 6:**
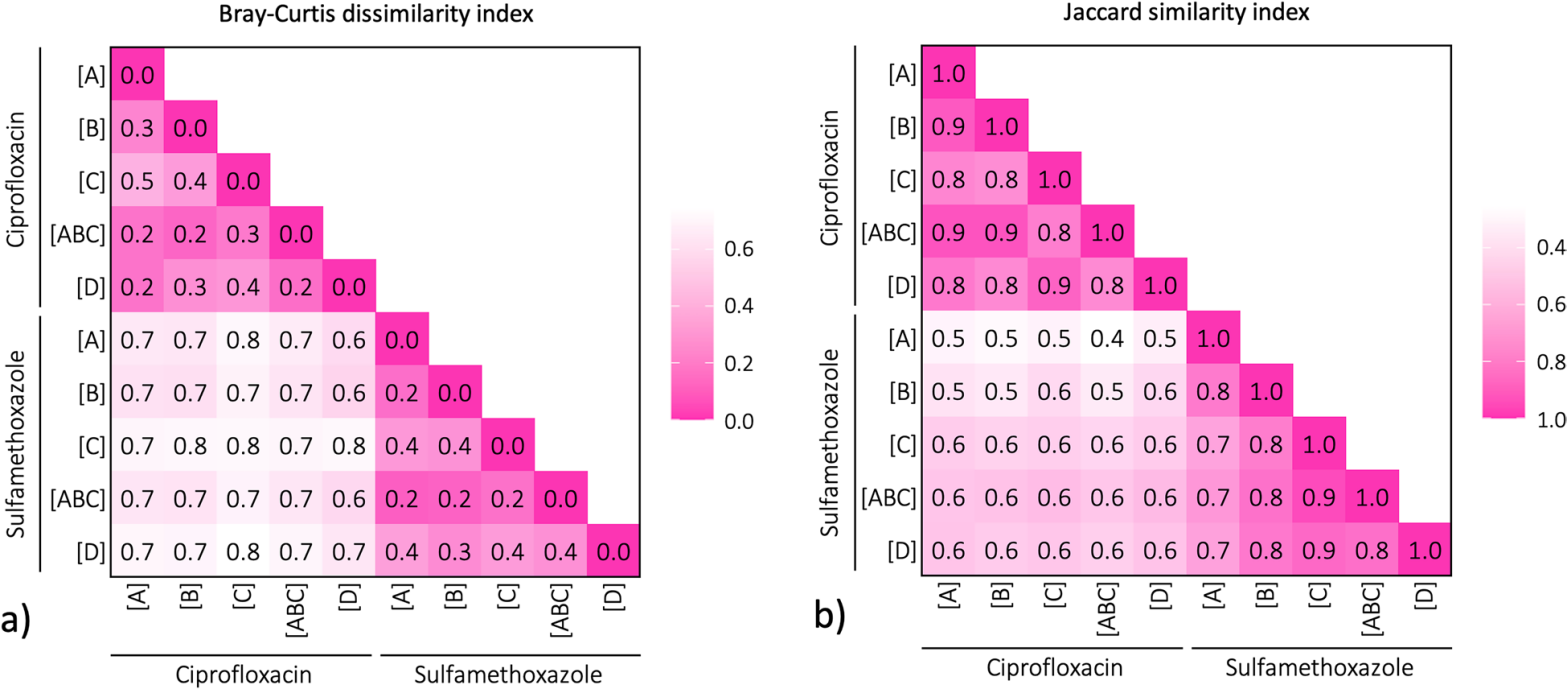
Heatmap matrices for the ciprofloxacin and sulfamethoxazole experiments representing the **a**) Bray–Curtis dissimilarity matrix and **b**) Jaccard similarity matrix. Dark pink shows sample sets with the highest similarity. For the Bray–Curtis dissimilarity indices (based on genus relative-abundance), 0.0 indicates complete similarity (identical sets) and 1.0 indicates no similarity (disjoint sets), whereas the opposite is true for the Jaccard similarity index (based on genus presence-absence) (i.e., 1.0 indicates complete similarity and 0.0 indicates no similarity)

The Bray–Curtis dissimilarity index, which measures differences in relative abundance between two sample sets, was calculated for each pair of samples as seen in Fig. 6a. Between-experiment dissimilarity (i.e., ciprofloxacin versus sulfamethoxazole) ranged from 0.6 to 0.8, suggesting a high level of genus composition dissimilarity, while within-experiment dissimilarity ranged from 0.2 to 0.5, indicating a moderate to substantial dissimilarity. The Jaccard similarity index, which determines the overlap of genus presence between sample sets, was calculated for each pair of samples as seen in Fig. 6b. Between-experiment similarity ranged from 0.4 to 0.6, suggesting a moderate level of genus overlap, whereas within-experiment similarity ranged from 0.7 to 0.9, indicating a relatively high level of overlap while still containing unique genera.

## Discussion

Despite the persistence of ciprofloxacin and sulfamethoxazole after 12 days in the BWDRs, the antibiotic degradation kinetics revealed notable decreases in their concentrations. These decreases likely resulted from mechanisms such as adsorption, chemisorption, biosorption, and/or biodegradation. The following sections consider within antibiotic experimental and between antibiotic experimental results valid for comparison given their community composition.

### Ciprofloxacin degradation kinetics

**Fig. 7:**
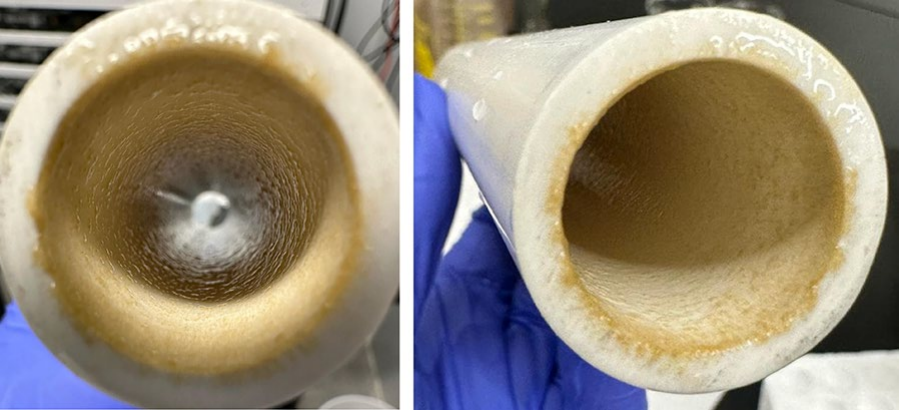
A removable section from the bench-top water distribution reactor (BWDR) PVC pipe showing the biofilm’s surface area coverage

Ciprofloxacin decreased in concentration by approximately 31.1% (± 3.9%) during exposure to biofilm and 27.4% (± 7.7%) in the PVC-only control. In the BWDRs with biofilm and the PVC-only control, it experienced a sharp initial decline and then reached a plateau after 1 day. Previous sorption kinetic studies have also found that ciprofloxacin quickly reaches a maximum uptake capacity before slowly equilibrating within a comparable timespan (e.g., 2 h, 3 h, and 6 h), irrespective of the experimental conditions (Maged et al. 2020; Atugoda et al. 2020; Wu et al. 2015). In Fig. 3a, the experimental replicates exposed to the biofilm appear to follow the same trend as the PVC-only control (i.e., no biofilm). This suggests that the ciprofloxacin was similarly sorbed to the PVC pipe material as it was to the biofilm, as all BWDRs had identical surface areas. A visual depiction of the biofilm surface area coverage can be found in Fig. 7. Previous studies have found that while ciprofloxacin had a low adsorption capacity for PVC, it had the highest adsorption capacity when compared to other antibiotics, such as sulfadiazine, which belongs to the same sulfonamide class as sulfamethoxazole (Li et al. 2018). Similarly, Donnelly (2011) found that while ciprofloxacin had a negligible decrease in concentration after 30 days, when kept in PVC minibags, it still decreased by 6.7% in concentration. Liu et al. (2019a, b) suggested that the slight adsorption of the hydrophilic ciprofloxacin may be due to its oxygen-containing functional groups reducing the hydrophobicity of the PVC. It was also found that as the concentration of ciprofloxacin in a solution increased, the amount of ciprofloxacin adsorbed to PVC also increased proportionally (Liu et al. 2019a).

Conversely, the trend observed in the experimental replicates with ciprofloxacin may be explained by the presence of extracellular polymeric substances (EPS) on the biofilms. EPS has been found to bind to almost all antibiotics (Matviichuk et al. 2023), thus limiting their penetration/diffusion into the matrix (Stewart 2015). In addition, at the neutral pH of water, the EPS will be negatively charged and partially hydrophilic (Bryers 2000). In this case, it has been found that between a pH of 6.1 to 8.7, ciprofloxacin is predominantly cationic (Wunder et al. 2011), facilitating the formation of binding EPS-ciprofloxacin complexes with the carboxyl, amine, and hydroxyl functional groups of the EPS (Zhang et al. 2018). Similar to the observations of the PVC sorption, Zhang et al. (2018) found that ciprofloxacin will reach an equilibrium after 20 h of exposure to EPS, likely due to exhaustion of adsorption sites on the EPS surface. If and once penetrated, the ciprofloxacin may have undergone biotransformation. Studies on degradation enzymes typically focus on the discovery of a single species (Liu et al. 2019b). Enzymes such as cytochrome P450, dehydrogenase, and protease (Fang et al. 2021; Wang et al. 2019) may have been present, contributing to the degradation kinetics.

In the context of DWDSs, these findings not only offer insight into the persistence of ciprofloxacin, but also shed light as to why it is detected at higher concentrations compared to other antibiotics. In essence, ciprofloxacin may saturate the substrates it interacts with (i.e., PVC, EPS) by reaching their sorption capacities while also depending on its level of concentration. Consequently, its concentration may then remain relatively constant, with the exception of open system dilutions. As PVC is projected to comprise 97% of DWDSs by the year 2100 (Wols and Thienen 2016) and the inevitability of multi-species biofilms forming along their surfaces (Rilstone et al. 2021), it is reasonable to anticipate that ciprofloxacin will not only continue to be detected, but may increase in concentration proportionally to its continued administration.

### Sulfamethoxazole degradation kinetics

Conversely, sulfamethoxazole decreased in concentration by approximately 87.2% (± 15.8%) during exposure to biofilm and 3.6% (± 8.6%) in the PVC-only control. When exposed to biofilm, it also had a sharp initial decline, continued to steadily decrease after 1 day, and then remained detectable after 12 days. Unlike ciprofloxacin, the PVC-only control and experimental replicates for sulfamethoxazole had substantially different reaction trends. In the PVC-only control, the concentration of sulfamethoxazole appeared to remain stable throughout the experiment, with a possible slight decrease after 1 day (Fig. 3b). However, this decrease was within the standard deviation error of the mean. Unlike ciprofloxacin that is positively charged at circumneutral pH, sulfamethoxazole is a neutral-to-negatively charged compound. This means that sulfamethoxazole could have a possible electrostatic discharge with the negatively charged PVC substrate (Wunder et al. 2011; Wang et al. 2023). Similarly, sulfamethoxazole is hydrophilic while PVC is hydrophobic (Wang et al. 2023). Over time, it is possible that the aging PVC could form hydrogen bonds with sulfamethoxazole as the main mechanism of adsorption (Wang et al. 2023). Previous abiotic sorption kinetic studies have found that sulfamethoxazole reaches a maximum adsorption capacity on PVC and comparable plastics between 16 to 24 h (Guo et al. 2019; Xu et al. 2018). However, these were observed at higher concentrations (i.e., 12 mg L^−1^ and 1 mg L^−1^, respectively). In this study, it is possible that the low concentration (i.e., 10 µg L^−1^) and 12-day contact time between the sulfamethoxazole and PVC was not sufficient to observe a delayed adsorption capacity.

Compared to the PVC-only control, the experimental replicates that contained biofilm exhibited a substantial decrease in sulfamethoxazole concentration. Li and Cui (2020) reported a similar reaction trend with sulfamethoxazole in estuarine water, where there was a rapid initial sorption followed by a relatively slow biodegradation. In terms of drinking water biofilm, this was likely due to the chemisorption that happens between the EPS and the sulfamethoxazole. More specifically, Pi et al. (2019) stated that the hydrophobic regions of the EPS increase their high binding strength to sulfamethoxazole and the presence of more adsorption sites on the EPS is facilitated by the weaker steric hindrance of side groups on sulfamethoxazole. Like in the present study, Pi et al. (2019) found a comparable biosorption efficiency of 70.0% in *Klebsiella* sp. J1 EPS. Thus far, no studies have been found to report whether sulfamethoxazole eventually reaches an equilibrium state when exposed to biofilm. In addition, it has been found that species of *Pseudomonas* and *Acinetobacter* are known to be sulfamethoxazole-degrading bacteria (Duc 2023). Enzymes such as peroxygenase and dioxygenase, (de Boer et al. 2025; Duc 2023) have been found to degrade sulfamethoxazole.

As previously mentioned, sulfamethoxazole is detected more frequently but at lower concentrations in DWDSs compared to ciprofloxacin. The sorption and possible subsequent degradation of sulfamethoxazole appears to be dependent on the substrate it interacts with, where it is more sensitive to biotic than abiotic factors. Despite the substantial decline in sulfamethoxazole concentration, it remained detectable after 12 days, consistent with the trends that have been reported in the literature (Rilstone et al. 2021). Whether sulfamethoxazole was detected in finished water may, therefore, be directly dependent on its retention time and the kind of substrates it is exposed to during that time.

### Biofilm TCC and antibiotic concentration correlations

As each antibiotic experiment was performed at a sub-MIC, no biofilms were found to have experienced a significant reduction in or inhibition of biofilm formation (Fig. 4). The only significant TCC growth rates were observed in the mean experimental replicates of the ciprofloxacin experiment, specifically between the overall time points, days 1 to 12, and when compared to the PVC-only control. However, it was found that despite this significance, no correlation was identified between the ciprofloxacin degradation rate to the TCC growth rate. Since correlative statistics do not imply causation (Altman and Krzywinski 2015), it is possible that while the ciprofloxacin degradation rate was independent of the TCCs, the TCCs may have been dependent on the ciprofloxacin degradation rate, resulting in their significant increase. Previous sub-MIC studies of ciprofloxacin have also reported enhanced biofilm formation (Whelan et al. 2022; Rafaque et al. 2020). Similarly, Gu et al. (2017) found that an increase in residual ciprofloxacin concentrations (i.e., 300 µg L^−1^) had a positive correlation with an increase in EPS.

Conversely, none of the TCC growth rates in the sulfamethoxazole experiment were significant and their lack of correlation with the sulfamethoxazole degradation rate was consistent with this finding. Upon closer inspection, it can be seen that while not significant, the TCCs were observed to decrease (Fig. 4b). While this decrease fell within the standard error of the mean and thus considered to be negligible, there is a possibility of an undetected dependent causation, particularly considering the significant rate of degradation of sulfamethoxazole with biofilm exposure. For instance, Brunchmann et al. (2013) found that sub-MICs of sulfamethoxazole resulted in an increase in biomass and thickness in *Pseudomonas aeruginosa* wastewater isolates.

For DWDSs, this raises concern for the possible effects that sub-MIC antibiotic degradations could have on the biofilms that line the pipe walls. If the residual antibiotic concentrations have an effect on the biofilm TCCs, then this could have ramifications for resistance promotion. For instance, prolonged exposure and/or degradation/sorption into the biofilm matrix could result in the promotion and development of antibiotic resistance genes (ARGs) and their subsequent dissemination.

### Antibiotic experiment comparisons

The genus community compositions within each antibiotic experiment (e.g., ciprofloxacin versus ciprofloxacin) had a moderate to substantial Bray–Curtis dissimilarity, meaning that even within the same experiment, the community compositions had a noticeable degree of difference in the relative abundance of their genera (Fig. 6a). Despite this, the experimental replicates for each antibiotic all had comparable reaction trends (Fig. 3). Between the antibiotic experiments (i.e., ciprofloxacin versus sulfamethoxazole), there was a high level of dissimilarity, however, this was likely exacerbated by the already moderate to substantial dissimilarity within the experiments. Therefore, the authors of this paper maintain that it is valid to compare each antibiotic experiment, as the observed dissimilarities fall within the expected variations. This was further validated by the Jaccard similarities that only consider the binary presence or absence of a genus. As seen in Fig. 6b, the within experiments had a high level of similarity while the between experiments had a moderate level of similarity, being closer in degree than the Bray–Curtis dissimilarities suggest. At the same time, there were a number of other genera that were not dominant and present in the “Others + unassigned” (Fig. 5) that would have influenced the indices. These results highlight the replicability and reproducibility of the effects that diverse multi-species microbial communities in DWDSs can have on antibiotic persistence.

### Impact of alternative parameters

The experiments were performed with a flow rate of 3 L min^−1^ (Reynolds number of 3000) and a corresponding wall shear stress of 0.125 Pa. It is unclear how a higher or lower shear stress might affect the degradation kinetics and the sorption of the antibiotics onto the biofilm. One possibility is that increasing the flow rate and wall shear stress might promote more EPS production in the biofilm to resist shear and, in doing so, provide added sites for the sorption of the antibiotics. Another possibility is that increasing the flow rate, Reynolds number, and shear stress might increase the level of turbulence in the pipe and further promote the movement of the antibiotics from the bulk water to the biofilm and further enhance adsorption. Further studies that examine the link between shear stress and antibiotic degradation are needed to answer these questions definitively.

Moreover, the selected concentration of the antibiotics was at a sub-MIC of 10 μg L^−1^. Increasing this concentration could surpass the sub-MIC threshold, potentially triggering specific metabolic pathways that enhance antibiotic degradation and sorption. Alternatively, even higher concentrations may exert inhibitory or bactericidal effects on the biofilm, reducing its ability to degrade or sorb the antibiotic. Conversely, decreasing the concentration may result in either the same or decreased rate of degradation. Further studies are needed to explore the effects of varying antibiotic concentrations.

In addition, the experiments had an average pH of 7.3, temperature of 21 °C, and biofilm TCC of approximately 250,000 cells cm^2^. The water quality parameters are within the typical ranges for both drinking water and wastewater (Liu et al. 2013). Each water quality parameter may accelerate the kinetic degradation differently. The pH may influence the ionization of the antibiotics, thus affecting their sorption to the biofilm. The temperature may influence the metabolic rate of the biofilm, likely increasing the rate of degradation and sorption. The biofilm TCC variances may provide more/less surface area for interactions and directly affect the rate of degradation based on the microbial activity. Further studies should be conducted to isolate and identify the combined effects of each of these parameters on each of the antibiotic degradation kinetics.

Furthermore, disinfectants in municipal systems such as chlorine, chloramine, and ozone may influence the degradation kinetics through direct oxidation or altering bacterial/biofilm structure and composition. Specifically, these disinfectants may generate reactive species, such as reactive chlorine species (RCS), increasing the rate of degradation kinetics through oxidation (Kim et al. 2020; Xu et al. 2022). However, there are several limitations to the antibiotic degradation kinetics by disinfection including excessive dosage leading to negative effects on removal rates, transformation by-products, and incomplete removal (Kim et al. 2020; Ruotong et al. 2024). Similarly, interactions with natural organic matter (NOM) and other water contaminants (e.g., heavy metals, other antibiotics) may influence the antibiotic degradation kinetics. For instance, NOM may either accelerate degradation by generating reactive species that enhance photolysis or decelerate it by forming complexes that inhibit photolysis (Song et al. 2021; Zhao et al. 2023). Likewise, other water contaminants may exhibit similar varied effects indirectly by affecting water quality parameters such as altering pH or microbial activity.

While the focus of this paper is on the degradation kinetics of antibiotics, a related study was undertaken by the authors to examine whether the same sub-MIC (10 μg L^−1^) of ciprofloxacin and sulfamethoxazole promote ARGs in drinking water biofilms (Rilstone et al. 2025a, 2025b). The results showed that while ciprofloxacin had no significant impact on ARG promotion, sulfamethoxazole did (Rilstone et al. 2025a, 2025b). Conversely, ciprofloxacin significantly impacted biofilm TCC, sulfamethoxazole did not (Rilstone et al. 2025a, 2025b). These results underscore the need for further investigation into the effects of antibiotic degradation kinetics on resistance promotion.

### Operational implications

Ciprofloxacin reached an equilibrium after exposure to the biofilm/EPS matrix likely due to the exhaustion of adsorption sites in the EPS layers. The implication is that ciprofloxacin has the potential to persist in bulk water at high residual concentrations. On the other hand, sulfamethoxazole was readily adsorbed to the biofilm/EPS matrix and as a result, it was presented in the bulk water at low concentrations. These findings underscore the importance of developing advanced treatment technologies and processes to effectively remove residual levels of antibiotics like ciprofloxacin and sulfamethoxazole that are found in source water of drinking water systems to achieve two operational goals: first, to avoid the risk of human ingestion of residual levels of antibiotics in drinking water at the tap, and; second to reduce the likelihood that resistance genes will be promoted and expressed where a high degree of antibiotic sorption takes place in the biofilm of the pipe.

## Conclusion

The degradation kinetics of the antibiotics ciprofloxacin and sulfamethoxazole and their effects on the TCCs of multi-species biofilms were investigated to better understand their ongoing persistence in DWDSs. When ciprofloxacin was exposed to biofilm, it did not exhibit a significant overall decline in concentration, except for the initial decrease noted at 10 min and 1 day, which were significant. The kinetic degradation of ciprofloxacin when exposed to biofilm followed a comparable rate of change to its PVC-only control that had no biofilm. Conversely, the TCCs in the biofilm exposed to ciprofloxacin did show a statistically significant increase. For sulfamethoxazole, there was a statistically significant decline at all time points, except for time 10 min to 1 day, when exposed to biofilm. The sulfamethoxazole PVC-only control showed no change when only exposed to PVC. Similarly, the TCCs in both the experimental replicates and biofilm control showed no significant changes. In the context of DWDSs, these results provide evidence as to why ciprofloxacin is detected less frequently, but at higher concentrations, whereas sulfamethoxazole is detected more frequently, but at lower concentrations. This study had a limited sample size and limited the investigation to the concentration of the parent compound of each antibiotic. The results obtained from this study fill the gap in antibiotic persistence research in three ways: first, it examines the persistence of commonly documented antibiotics; second, it examines their persistence over a 12 day extended period; and third it examines antibiotic persistence in a fully-controlled environment. The findings of the paper underscore the importance of developing advanced treatment technologies and processes to effectively remove residual levels of antibiotics to avoid the risk of human ingestion of antibiotics at the tap, and to reduce the likelihood of antibiotics being adsorbed to the biofilm and promoting antimicrobial resistance in the biofilm.

## Supplementary Information

Supplementary material 1.

## Data Availability

No datasets were generated or analysed during the current study.
