# Supplementary material for: Study on the persistence of ciprofloxacin and sulfamethoxazole in simulated drinking water systems
Source: Environ Syst Res (Heidelb). 2025 Apr 26;14(1):7. doi: 10.1186/s40068-025-00396-5 (PMC12033125; doi:10.1186/s40068-025-00396-5)
Supplement: Supplementary file 1 — Supplementary material 1. [file 40068_2025_396_MOESM1_ESM.docx]

**Supplementary Material**

**Table S1.** Water quality parameters.

|  |  |  | **Bulk Water** | | | | | | | | | **Coupon** | |
| --- | --- | --- | --- | --- | --- | --- | --- | --- | --- | --- | --- | --- | --- |
| **Antibiotic** | **Week** | **Name** | **Temperature**  **(°C)** | **pH** | **Turbidity**  **(NTU)** | **UV absorb at 600 nm**  **(A)** | **TOC**  **(mg** $\boldsymbol{\cdot}$ **L^-1^)** | **TN**  **(mg** $\boldsymbol{\cdot}$ **L^-1^)** | **Free Cl**  **(mg** $\boldsymbol{\cdot}$ **L^-1^)** | **ATP**  **(pg** $\boldsymbol{\cdot}$ **mL^-1^)** | **TCC**  **(cell** $\boldsymbol{\cdot}$ **mL^-1^)** | **ATP**  **(pg** $\boldsymbol{\cdot}$ **cm^2^)** | **TCC**  **(cell** $\boldsymbol{\cdot}$ **cm^2^)** |
| Ciprofloxacin | 0 | GAC | 20.8 | 7.25 | 0.29 | 0.00 | 2.03 | 0.41 | 0.02 | 5 | 395 |  |  |
|  | 1 | A | 21.3 | 7.12 | 19.9 | 0.06 | 10.00 | 12.56 |  | 40,074 | 99300 |  |  |
|  |  | B | 21.2 | 7.43 | 23.9 | 0.07 | 11.38 | 13.09 |  | 35,359 | 98615 |  |  |
|  |  | C | 21.1 | 7.65 | 22.2 | 0.08 | 10.28 | 13.25 |  | 31,607 | 100944 |  |  |
|  |  | D | 20.8 | 7.49 | 18.1 | 0.06 | 9.81 | 14.14 |  | 25,416 | 74852 |  |  |
|  | 2 | GAC | 20.8 |  | 0.18 | 0.00 | 1.87 | 0.42 | 0.02 | 6 | 629 |  |  |
|  |  | A | 21.3 | 7.12 | 19.9 | 0.06 | 10 | 12.56 |  | 40,074 | 99300 | 30,626 | 83,355 |
|  |  | B | 21.2 | 7.43 | 23.9 | 0.07 | 11.38 | 13.09 |  | 35,359 | 98615 | 15,935 | 58,276 |
|  |  | C | 21.1 | 7.65 | 22.2 | 0.08 | 10.28 | 13.25 |  | 31,607 | 100944 | 2,711 | 16,003 |
|  |  | D | 20.8 | 7.49 | 18.1 | 0.06 | 9.81 | 14.14 |  | 25,416 | 74852 | 13,424 | 32,949 |
|  | 3 | A | 21.3 | 7.33 | 16.7 | 0.06 | 8.70 | 12.99 |  | 21,205 | 92,943 |  |  |
|  |  | B | 21.2 | 7.38 | 15 | 0.06 | 8.36 | 12.98 |  | 27,422 | 169,987 |  |  |
|  |  | C | 21.1 | 7.03 | 12.9 | 0.05 | 9.12 | 12.62 |  | 31,731 | 201,931 |  |  |
|  |  | D | 20.8 | 7.49 | 15.8 | 0.06 | 8.47 | 12.22 |  | 29,934 | 109,980 |  |  |
|  | 4 | GAC | 20.8 | 7.66 | 0.29 | 0.00 | 2.28 | 0.47 | 0.02 | 113 | 14731 |  |  |
|  |  | A | 21.3 | 7.44 | 29.2 | 0.09 | 14.70 | 27.09 |  | 38,233 | 253,953 | 9,515 | 65,327 |
|  |  | B | 21.2 | 7.42 | 24.5 | 0.08 | 15.53 | 25.96 |  | 36,517 | 227,476 | 10,762 | 19,024 |
|  |  | C | 21.1 | 7.02 | 28.9 | 0.10 | 18.68 | 26.22 |  | 37,913 | 94,642 | 5,952 | 10,883 |
|  |  | D | 20.8 | 7.17 | 31.1 | 0.11 | 16.77 | 26.00 |  | 39,566 | 725,282 | 1,601 | 21,927 |
|  | 5 | A | 21.3 | 7.25 | 8.2 | 0.03 | 8.69 | 14.49 |  | 11,423 | 170,534 |  |  |
|  |  | B | 21.2 | 7.19 | 18.9 | 0.07 | 8.83 | 12.16 |  | 34,227 | 85,405 |  |  |
|  |  | C | 21.1 | 7.19 | 11.8 | 0.04 | 10.90 | 11.60 |  | 23,329 | 958,200 |  |  |
|  |  | D | 20.8 | 7.26 | 18.6 | 0.07 | 9.23 | 12.89 |  | 23,052 | 210,434 |  |  |
|  | 6 | GAC | 20.8 | 7.55 | 0.18 | 0.00 | 1.93 | 0.43 | 0.02 | 6 | 293 |  |  |
|  |  | A | 21.3 | 7.45 | 11.8 | 0.03 | 15.80 | 32.14 |  | 14,304 | 205,901 | 8,982 | 116,673 |
|  |  | B | 21.2 | 7.33 | 29.1 | 0.09 | 15.63 | 28.87 |  | 37,190 | 442,719 | 8,708 | 51,634 |
|  |  | C | 21.1 | 7.24 | 26.2 | 0.08 | 20.46 | 25.73 |  | 27,748 | 90,737 | 19,702 | 46,036 |
|  |  | D | 20.8 | 7.31 | 29.2 | 0.10 | 16.14 | 27.31 |  | 34,584 | 335,136 | 778 | 17,079 |
|  | 7 | A | 21.3 | 7.27 | 10.6 | 0.03 | 8.98 | 15.58 |  | 19,723 | 56,897 |  |  |
|  |  | B | 21.2 | 7.39 | 13.7 | 0.05 | 9.26 | 15.67 |  | 29,370 | 171,684 |  |  |
|  |  | C | 21.1 | 7.08 | 18.8 | 0.06 | 9.74 | 16.81 |  | 31,768 | 130,231 |  |  |
|  |  | D | 20.8 | 7.21 | 17.9 | 0.06 | 8.82 | 14.45 |  | 34,586 | 127,697 |  |  |
|  | 8 | GAC | 20.8 | 7.62 | 0.14 | 0.00 | 1.78 | 0.46 | 0.02 | 15 | 2458 |  |  |
|  |  | A | 21.3 | 7.25 | 25.7 | 0.08 | 15.08 | 30.12 |  | 31,322 | 38,633 | 38,956 | 1,86,800 |
|  |  | B | 21.2 | 7.31 | 26.7 | 0.09 | 16.02 | 30.65 |  | 48,470 | 6,451,613 | 1,360 | 25,803 |
|  |  | C | 21.1 | 7.31 | 34.8 | 0.10 | 17.38 | 32.91 |  | 41,543 | 184,387 | 10,551 | 24,858 |
|  |  | D | 20.8 | 7.34 | 31.4 | 0.10 | 19.58 | 28.72 |  | 48,637 | 96,426 | 3,628 | 16,886 |
|  | 9 | A | 21.3 | 7.25 | 4.53 | 0.01 | 8.38 | 17.46 |  | 8,267 | 382,046 |  |  |
|  |  | B | 21.2 | 7.35 | 15 | 0.05 | 10.20 | 15.96 |  | 32,380 | 241,130 |  |  |
|  |  | C | 21.1 | 7.3 | 14.4 | 0.05 | 10.31 | 15.62 |  | 27,540 | 286,302 |  |  |
|  |  | D | 20.8 | 7.54 | 22 | 0.07 | 10.20 | 14.05 |  | 39,083 | 30,082 |  |  |
|  | 10 | GAC | 20.8 | 7.39 | 0.13 | 0.00 | 2.03 | 0.48 | 0.02 | 6 | 504 |  |  |
|  |  | A | 21.3 | 7.23 | 14.4 | 0.05 | 16.07 | 34.53 |  | 28,353 | 978,258 | 21,923 | 404,741 |
|  |  | B | 21.2 | 7.35 | 25.2 | 0.08 | 18.61 | 32.82 |  | 46,051 | 206,734 | 3,612 | 12,279 |
|  |  | C | 21.1 | 7.24 | 32.4 | 0.10 | 18.24 | 31.53 |  | 53,596 | 128,526 | 340 | 8,943 |
|  |  | D | 20.8 | 7.33 | 34.4 | 0.10 | 28.97 | 29.52 |  | 52,006 | 53,979 | 3,699 | 19,712 |
|  | 11 | A | 21.3 | 7.14 | 5.36 | 0.02 | 14.58 | 19.76 |  | 9,531.35 | 1,197,973 |  |  |
|  |  | B | 21.2 | 7.22 | 16.2 | 0.06 | 12.04 | 17.08 |  | 28,797.53 | 39,806 |  |  |
|  |  | C | 21.1 | 7.09 | 13.2 | 0.04 | 10.72 | 17.12 |  | 22,685.27 | 78,114 |  |  |
|  |  | D | 20.8 | 7.27 | 10 | 0.03 | 13.99 | 14.79 |  | 22,330.10 | 5,496 |  |  |
|  | 12 | GAC | 20.8 | 7.27 | 0.17 | 0.00 | 2.26 | 0.53 | 0.02 | 5.46 | 252 |  |  |
|  |  | A | 21.3 | 7.43 | 17.4 | 0.06 | 18.25 | 31.73 |  | 32,502.01 | 455,897 | 40,547.26 | 5,20,210 |
|  |  | B | 21.2 | 7.45 | 27.8 | 0.09 | 20.93 | 32.87 |  | 48,453.44 | 33,056 | 1,154.97 | 11,944 |
|  |  | C | 21.1 | 7.3 | 26.7 | 0.08 | 19.20 | 34.09 |  | 47,766.28 | 2,32,779 | 50,161.69 | 19,871 |
|  |  | D | 20.8 | 7.37 | 23.6 | 0.07 | 42.72 | 32.46 |  | 36,904.08 | 20,045 | 13,858.69 | 16,636 |
|  | 12.1 | A_1_ |  |  |  |  |  |  |  | 24,449.00 | 77,422 | 142,427.78 | 639,716 |
|  |  | A_2_ |  |  |  |  |  |  |  |  |  |  | 1,038,725 |
|  |  | A_3_ |  |  |  |  |  |  |  |  |  |  | 864,102 |
|  |  | B_1_ |  |  |  |  |  |  |  | 33,624.03 | 29,694 | 1,063.53 | 6,049 |
|  |  | B_2_ |  |  |  |  |  |  |  |  |  |  | 11,463 |
|  |  | B_3_ |  |  |  |  |  |  |  |  |  |  | 10,951 |
|  |  | C_1_ |  |  |  |  |  |  |  | 19,320.71 | 121,883 | 46,538.16 | 9,944 |
|  |  | C_2_ |  |  |  |  |  |  |  |  |  |  | 14,570 |
|  |  | C_3_ |  |  |  |  |  |  |  |  |  |  | 12,356 |
|  |  | D_1_ |  |  |  |  |  |  |  | 40,061.27 | 50613 | 11,575.45 | 32,146 |
|  |  | D_2_ |  |  |  |  |  |  |  |  |  |  | 23,324 |
|  |  | D_3_ |  |  |  |  |  |  |  |  |  |  | 21,841 |
|  | 12.2 | A_1_ |  |  |  |  |  |  |  | 25,714.96 | 41,642 | 75,185.57 | 627,567 |
|  |  | A_2_ |  |  |  |  |  |  |  |  |  |  | 520,516 |
|  |  | A_3_ |  |  |  |  |  |  |  |  |  |  | 838,325 |
|  |  | B_1_ |  |  |  |  |  |  |  | 26,103.02 | 37197 | 8,236.79 | 6,595 |
|  |  | B_2_ |  |  |  |  |  |  |  |  |  |  | 18,537 |
|  |  | B_3_ |  |  |  |  |  |  |  |  |  |  | 9,506 |
|  |  | C_1_ |  |  |  |  |  |  |  | 17,928.09 | 16,021 | 17,959.09 | 17,518 |
|  |  | C_2_ |  |  |  |  |  |  |  |  |  |  | 50,106 |
|  |  | C_3_ |  |  |  |  |  |  |  |  |  |  | 144,800 |
|  |  | D_1_ |  |  |  |  |  |  |  | 32,493.42 | 27,942 | 10,658.74 | 46,182 |
|  |  | D_2_ |  |  |  |  |  |  |  |  |  |  | 19,264 |
|  |  | D_3_ |  |  |  |  |  |  |  |  |  |  | 39,036 |
|  | 13 | A |  | 7.02 | 5.59 | 0.03 | 9.91 | 19.89 |  | 16,376.28 | 1,289,932 |  |  |
|  |  | B |  | 7.13 | 15.1 | 0.05 | 11.96 | 16.69 |  | 23,995.39 | 55,084 |  |  |
|  |  | C |  | 7.3 | 3.34 | 0.01 | 12.11 | 18.37 |  | 2,676.18 | 322,764 |  |  |
|  |  | D |  | 7.25 | 31.4 | 0.09 | 12.12 | 16.92 |  | 38,180.74 | 37,453 |  |  |
|  | 14 | GAC | 20.8 | 7.52 | 0.15 | 0.00 | 2.53 | 0.46 | 0.02 | 5.55 | 1928 |  |  |
|  |  | A_1_ | 21.3 | 7.15 | 12 | 0.05 | 17.74 | 37.90 |  | 26,685.55 | 1,372,457 | 19,790.58 | 3,351,313 |
|  |  | A_2_ |  |  |  |  |  |  |  |  |  |  | 1,106,926 |
|  |  | A_3_ |  |  |  |  |  |  |  |  |  |  | 3,107,879 |
|  |  | B_1_ | 21.2 | 7.08 | 32.9 | 0.09 | 21.38 | 34.19 |  | 41,589.55 | 131,414 | 1,393.21 | 33,023 |
|  |  | B_2_ |  |  |  |  |  |  |  |  |  |  | 24,658 |
|  |  | B_3_ |  |  |  |  |  |  |  |  |  |  | 20,345 |
|  |  | C_1_ | 21.1 | 7.21 | 7.07 | 0.03 | 20.55 | 38.64 |  | 9,339.59 | 1,094,408 | 13,642.72 | 556,113 |
|  |  | C_2_ |  |  |  |  |  |  |  |  |  |  | 507,636 |
|  |  | C_3_ |  |  |  |  |  |  |  |  |  |  | 932,232 |
|  |  | D_1_ | 20.8 | 7.21 | 32.1 | 0.09 | 26.82 | 33.06 |  | 40,535.36 | 63,950 | 3,689.28 | 31,736 |
|  |  | D_2_ |  |  |  |  |  |  |  |  |  |  | 22,231 |
|  |  | D_3_ |  |  |  |  |  |  |  |  |  |  | 41,183 |
| Sulfamethoxazole | 0 | GAC | 20.8 | 7.45 | 0.12 | 0 | 1.83 | 0.446 | 0.02 | 4 | 224 |  |  |
|  | 2 | GAC | 20.8 | 7.51 |  |  | 2.21 | 0.41 | 0.02 | 12 | 1273 |  |  |
|  |  | A_1_ |  | 7.27 |  |  | 25.79 | 25.38 |  | 75,449.22 | 746,820 |  |  |
|  |  | A_2_ |  |  |  |  |  |  |  |  |  |  |  |
|  |  | A_3_ |  |  |  |  |  |  |  |  |  |  |  |
|  |  | B_1_ |  | 7.24 |  |  | 38.22 | 27.51 |  | 72,332.43 | 122,932 |  |  |
|  |  | B_2_ |  |  |  |  |  |  |  |  |  |  |  |
|  |  | B_3_ |  |  |  |  |  |  |  |  |  |  |  |
|  |  | C_1_ |  | 7.06 |  |  | 19.03 | 27.28 |  | 76,904.56 | 447,564 |  |  |
|  |  | C_2_ |  |  |  |  |  |  |  |  |  |  |  |
|  |  | C_3_ |  |  |  |  |  |  |  |  |  |  |  |
|  |  | D_1_ |  | 7.02 |  |  | 37.59 | 28.13 |  | 80,454.34 | 626,280 |  |  |
|  |  | D_2_ |  |  |  |  |  |  |  |  |  |  |  |
|  |  | D_3_ |  |  |  |  |  |  |  |  |  |  |  |
|  | 4 | GAC |  | 7.71 |  |  | 1.72 | 0.43 |  | 17.53 | 1837 |  |  |
|  |  | A_1_ |  | 7.81 |  |  | 16.25 | 29.59 |  | 66,175.10 | 134,638 | 29,958.32 | 22,278 |
|  |  | A_2_ |  |  |  |  |  |  |  |  |  |  | 23,463 |
|  |  | A_3_ |  |  |  |  |  |  |  |  |  |  | 10,537 |
|  |  | B_1_ |  | 7.55 |  |  | 16.5 | 27.39 |  | 74,393.47 | 30,645 | 18,964.79 | 17,640 |
|  |  | B_2_ |  |  |  |  |  |  |  |  |  |  | 25,261 |
|  |  | B_3_ |  |  |  |  |  |  |  |  |  |  | 21,906 |
|  |  | C_1_ |  | 7.3 |  |  | 19 | 28.43 |  | 66,401.08 | 109,703 | 5,019.32 | 10,271 |
|  |  | C_2_ |  |  |  |  |  |  |  |  |  |  | 24,834 |
|  |  | C_3_ |  |  |  |  |  |  |  |  |  |  | 16,541 |
|  |  | D_1_ |  | 7.19 |  |  | 16.48 | 27.16 |  | 48,902.95 | 153,262 | 24,025.52 | 17,813 |
|  |  | D_2_ |  |  |  |  |  |  |  |  |  |  | 24,024 |
|  |  | D_3_ |  |  |  |  |  |  |  |  |  |  | 22,239 |
|  | 6 | GAC |  | 7.49 |  |  | 2.08 | 0.53 |  | 12.72 | 192 |  |  |
|  |  | A_1_ |  | 7.44 |  |  | 17.4 | 28.58 |  | 42,282.42 | 201,435 |  |  |
|  |  | A_2_ |  |  |  |  |  |  |  |  |  |  |  |
|  |  | A_3_ |  |  |  |  |  |  |  |  |  |  |  |
|  |  | B_1_ |  | 7.43 |  |  | 16.69 | 28.07 |  | 44,913.12 | 151,418 |  |  |
|  |  | B_2_ |  |  |  |  |  |  |  |  |  |  |  |
|  |  | B_3_ |  |  |  |  |  |  |  |  |  |  |  |
|  |  | C_1_ |  | 7.23 |  |  | 18.03 | 28.64 |  | 46,197.38 | 72,523 |  |  |
|  |  | C_2_ |  |  |  |  |  |  |  |  |  |  |  |
|  |  | C_3_ |  |  |  |  |  |  |  |  |  |  |  |
|  |  | D_1_ |  | 7.08 |  |  | 16.69 | 23.76 |  | 34,010.13 | 55,638 |  |  |
|  |  | D_2_ |  |  |  |  |  |  |  |  |  |  |  |
|  |  | D_3_ |  |  |  |  |  |  |  |  |  |  |  |
|  | 8 | GAC |  | 7.49 |  |  | 1.71 | 0.43 |  | 8.57 | 122 |  |  |
|  |  | A_1_ |  | 7.09 |  |  | 16.82 | 25.05 |  | 41002 | 104,772.17 | 104,772.17 | 132,297 |
|  |  | A_2_ |  |  |  |  |  |  |  |  |  |  | 156,723 |
|  |  | A_3_ |  |  |  |  |  |  |  |  |  |  | 135,629 |
|  |  | B_1_ |  | 7.26 |  |  | 17.71 | 31.57 |  | 36,511.83 | 191,277 | 24,565.58 | 58,921 |
|  |  | B_2_ |  |  |  |  |  |  |  |  |  |  | 53,505 |
|  |  | B_3_ |  |  |  |  |  |  |  |  |  |  | 57,573 |
|  |  | C_1_ |  | 7.12 |  |  | 17.92 | 26.86 |  | 43,631.01 | 34,136 | 37,419.69 | 28,349 |
|  |  | C_2_ |  |  |  |  |  |  |  |  |  |  | 4,180 |
|  |  | C_3_ |  |  |  |  |  |  |  |  |  |  | 32,810 |
|  |  | D_1_ |  | 6.97 |  |  | 17.33 | 27.83 |  | 39,692.25 | 51,477 | 69,373.71 | 91,329 |
|  |  | D_2_ |  |  |  |  |  |  |  |  |  |  | 74,387 |
|  |  | D_3_ |  |  |  |  |  |  |  |  |  |  | 121,079 |
|  | 10 | GAC |  | 7.29 |  |  | 1.95 | 0.72 |  | 66.43 | 5169 |  |  |
|  |  | A_1_ |  | 7.11 |  |  | 17.5 | 28.91 |  | 45,341.74 | 61,830 |  |  |
|  |  | A_2_ |  |  |  |  |  |  |  |  |  |  |  |
|  |  | A_3_ |  |  |  |  |  |  |  |  |  |  |  |
|  |  | B_1_ |  | 7.22 |  |  | 17.92 | 26.54 |  | 32,948.65 | 64,671 |  |  |
|  |  | B_2_ |  |  |  |  |  |  |  |  |  |  |  |
|  |  | B_3_ |  |  |  |  |  |  |  |  |  |  |  |
|  |  | C_1_ |  | 7.16 |  |  | 24.62 | 28.11 |  | 33,146.58 | 46,712 |  |  |
|  |  | C_2_ |  |  |  |  |  |  |  |  |  |  |  |
|  |  | C_3_ |  |  |  |  |  |  |  |  |  |  |  |
|  |  | D_1_ |  | 7.03 |  |  | 17.77 | 31.63 |  | 42,561.23 | 68,737 |  |  |
|  |  | D_2_ |  |  |  |  |  |  |  |  |  |  |  |
|  |  | D_3_ |  |  |  |  |  |  |  |  |  |  |  |
|  | 12 | GAC |  | 7.85 |  |  | 1.79 | 0.46 |  | 21.38 | 638 |  |  |
|  |  | A_1_ |  | 6.88 |  |  | 16.12 | 30.1 |  | 39,992.17 | 117,211 | 97,125.00 | 208,106 |
|  |  | A_2_ |  |  |  |  |  |  |  |  |  |  | 105,671 |
|  |  | A_3_ |  |  |  |  |  |  |  |  |  |  | 119,931 |
|  |  | B_1_ |  | 7.12 |  |  | 45.52 | 32.41 |  | 80,093.60 | 124,245 | 11,826.13 | 9,830 |
|  |  | B_2_ |  |  |  |  |  |  |  |  |  |  | 43,861 |
|  |  | B_3_ |  |  |  |  |  |  |  |  |  |  | 43,015 |
|  |  | C_1_ |  | 7.1 |  |  | 22.38 | 29.2 |  | 43,464.56 | 69,764 | 8,597.93 | 66,730 |
|  |  | C_2_ |  |  |  |  |  |  |  |  |  |  | 34,701 |
|  |  | C_3_ |  |  |  |  |  |  |  |  |  |  | 94,048 |
|  |  | D_1_ |  | 6.85 |  |  | 32.59 | 30.92 |  | 67,579.44 | 94,062 | 87,941.78 | 153,415 |
|  |  | D_2_ |  |  |  |  |  |  |  |  |  |  | 149,271 |
|  |  | D_3_ |  |  |  |  |  |  |  |  |  |  | 203,515 |
|  | 12.1 | A_1_ |  |  |  |  |  |  |  | 39,627.70 | 61,893 | 374,592.28 | 198,617 |
|  |  | A_2_ |  |  |  |  |  |  |  |  |  |  | 498,910 |
|  |  | A_3_ |  |  |  |  |  |  |  |  |  |  | 350,844 |
|  |  | B_1_ |  |  |  |  |  |  |  | 17,991.57 | 28,529 | 124,715.64 | 106,092 |
|  |  | B_2_ |  |  |  |  |  |  |  |  |  |  | 83,532 |
|  |  | B_3_ |  |  |  |  |  |  |  |  |  |  | 36,476 |
|  |  | C_1_ |  |  |  |  |  |  |  | 30,471.40 | 113,830 | 8,586.65 | 34,984 |
|  |  | C_2_ |  |  |  |  |  |  |  |  |  |  | 63,504 |
|  |  | C_3_ |  |  |  |  |  |  |  |  |  |  | 460,277 |
|  |  | D_1_ |  |  |  |  |  |  |  | 26,145.83 | 40,004 | 378,124.08 | 125,718 |
|  |  | D_2_ |  |  |  |  |  |  |  |  |  |  | 477,910 |
|  |  | D_3_ |  |  |  |  |  |  |  |  |  |  | 287,956 |
|  | 12.2 | A_1_ |  |  |  |  |  |  |  | 28,926.43 | 43,792 | 311,413.46 | 227,314 |
|  |  | A_2_ |  |  |  |  |  |  |  |  |  |  | 238,155 |
|  |  | A_3_ |  |  |  |  |  |  |  |  |  |  | 342,385 |
|  |  | B_1_ |  |  |  |  |  |  |  | 29,360.52 | 36,375 | 184,412.54 | 195702 |
|  |  | B_2_ |  |  |  |  |  |  |  |  |  |  | 102670 |
|  |  | B_3_ |  |  |  |  |  |  |  |  |  |  | 132195 |
|  |  | C_1_ |  |  |  |  |  |  |  | 35,341.15 | 123,063 | 135,698.92 | 92,747 |
|  |  | C_2_ |  |  |  |  |  |  |  |  |  |  | 46,709 |
|  |  | C_3_ |  |  |  |  |  |  |  |  |  |  | 23,813 |
|  |  | D_1_ |  |  |  |  |  |  |  | 28,065.81 | 36,280 | 342,389.22 | 228,372 |
|  |  | D_2_ |  |  |  |  |  |  |  |  |  |  | 340,837 |
|  |  | D_3_ |  |  |  |  |  |  |  |  |  |  | 185,147 |
|  | 14 | GAC |  | 7.45 |  |  | 2.08 | 0.50 |  | 4.54 | 691 |  |  |
|  |  | A_1_ |  | 7.3 |  |  | 16.66 | 34.9 |  | 71,047.90 | 108,102 | 44,781.43 | 105,708 |
|  |  | A_2_ |  |  |  |  |  |  |  |  |  |  | 85,370 |
|  |  | A_3_ |  |  |  |  |  |  |  |  |  |  | 89,467 |
|  |  | B_1_ |  | 7.15 |  |  | 20.36 | 29.85 |  | 31,924.48 | 52,980 | 55,812.32 | 9,985 |
|  |  | B_2_ |  |  |  |  |  |  |  |  |  |  | 12,717 |
|  |  | B_3_ |  |  |  |  |  |  |  |  |  |  | 221,393 |
|  |  | C_1_ |  | 7.29 |  |  | 25.66 | 39.34 |  | 39,780.52 | 256,916 | 2,476.78 | 11,090 |
|  |  | C_2_ |  |  |  |  |  |  |  |  |  |  | 26,138 |
|  |  | C_3_ |  |  |  |  |  |  |  |  |  |  | 17,929 |
|  |  | D_1_ |  | 6.98 |  |  | 21.35 | 31.82 |  | 39,978.29 | 78,215 | 61,326.79 | 193,513 |
|  |  | D_2_ |  |  |  |  |  |  |  |  |  |  | 140,249 |
|  |  | D_3_ |  |  |  |  |  |  |  |  |  |  | 155,703 |

**Table S2.** Antibiotic concentration raw data for kinetic degradations. Sample ID column represents the specific order that samples were taken in.

| Antibiotic | Time | Sample ID | [A] | [B] | [C] | [ABC] | [X] |
| --- | --- | --- | --- | --- | --- | --- | --- |
| Ciprofloxacin | 0.0 | 1 | 10.000 | 10.000 | 10.000 | 10.000 | 10.000 |
|  |  | 2 | 10.000 | 10.000 | 10.000 | 10.000 | 10.000 |
|  |  | 3 | 10.000 | 10.000 | 10.000 | 10.000 | 10.000 |
|  | 0.1 | 1 | 8.648 | 8.160 | 8.151 | 8.320 | 8.459 |
|  |  | 2 | 8.775 | 8.913 | 8.263 | 8.650 | 9.087 |
|  |  | 3 | 8.976 | 9.034 | 8.403 | 8.804 | 9.322 |
|  | 1.0 | 1 | 7.607 | 6.867 | 6.069 | 6.848 | 7.384 |
|  |  | 2 | 7.770 | 8.225 | 7.008 | 7.668 | 8.631 |
|  |  | 3 | 8.063 | 8.294 | 7.042 | 7.800 | 8.642 |
|  | 6.0 | 1 | 5.784 | 6.808 | 5.217 | 5.936 | 6.131 |
|  |  | 2 | 6.806 | 6.941 | 6.581 | 6.776 | 8.017 |
|  |  | 3 | 6.968 | 7.023 | 7.450 | 7.147 | 8.716 |
|  | 12.0 | 1 | 6.151 | 6.505 | 6.874 | 6.510 | 6.278 |
|  |  | 2 | 6.262 | 6.631 | 7.322 | 6.738 | 6.653 |
|  |  | 3 | 7.049 | 7.631 | 7.592 | 7.424 | 7.754 |
| Sulfamethoxazole | 0.0 | 1 | 10.000 | 10.000 | 10.000 | 10.000 | 10.000 |
|  |  | 2 | 10.000 | 10.000 | 10.000 | 10.000 | 10.000 |
|  |  | 3 | 10.000 | 10.000 | 10.000 | 10.000 | 10.000 |
|  | 0.1 | 1 | 5.835 | 5.623 | 5.605 | 5.688 | 7.86 |
|  |  | 2 | 6.114 | 5.955 | 5.179 | 5.749 | 11.22 |
|  |  | 3 | 5.103 | 5.998 | 5.277 | 5.460 | 11.10 |
|  | 1.0 | 1 | 5.028 | 5.233 | 5.581 | 5.281 | 8.44 |
|  |  | 2 | 5.891 | 5.675 | 5.738 | 5.768 | 10.15 |
|  |  | 3 | 5.783 | 5.587 | 5.777 | 5.716 | 10.59 |
|  | 6.0 | 1 | 4.642 | 3.267 | 3.912 | 3.940 | 7.65 |
|  |  | 2 | 5.119 | 3.587 | 2.769 | 3.825 | 11.43 |
|  |  | 3 | 5.214 | 3.589 | 4.407 | 4.403 | 10.25 |
|  | 12.0 | 1 | 3.054 | 0.150 | 0.605 | 1.270 | 8.78 |
|  |  | 2 | 3.071 | 0.150 | 0.620 | 1.280 | 9.64 |
|  |  | 3 | 3.126 | 0.150 | 0.617 | 1.297 | 10.50 |

**Table S3.** Calculated antibiotic kinetic degradation rates of change, where Δt represents change in time in days. Sample ID column represents the specific order that samples were taken from, corresponding with Table S2.

| Antibiotic | Δt (days) | Sample ID | Δ[A] | Δ[B] | Δ[C] | Δ[ABC] | Δ[X] |
| --- | --- | --- | --- | --- | --- | --- | --- |
| Ciprofloxacin | 0 to 0.1 | 1 | -13.520 | -18.400 | -18.490 | -16.803 | -15.410 |
|  |  | 2 | -12.250 | -10.870 | -17.370 | -13.497 | -9.130 |
|  |  | 3 | -10.240 | -9.660 | -15.970 | -11.957 | -6.780 |
|  | 0.1 to 1.0 | 1 | -1.157 | -1.437 | -2.313 | -1.636 | -1.194 |
|  |  | 2 | -1.117 | -0.764 | -1.394 | -1.092 | -0.507 |
|  |  | 3 | -1.014 | -0.822 | -1.512 | -1.116 | -0.756 |
|  | 1.0 to 6.0 | 1 | -0.365 | -0.012 | -0.170 | -0.182 | -0.251 |
|  |  | 2 | -0.193 | -0.257 | -0.085 | -0.178 | -0.123 |
|  |  | 3 | -0.219 | -0.254 | 0.082 | -0.131 | 0.015 |
|  | 6.0 to 12.0 | 1 | -0.365 | -0.051 | 0.276 | 0.096 | 0.025 |
|  |  | 2 | -0.193 | -0.052 | 0.124 | -0.006 | -0.227 |
|  |  | 3 | -0.219 | 0.101 | 0.024 | 0.046 | -0.160 |
|  | 0.0 to 12.0 | 1 | -0.321 | -0.291 | -0.261 | -0.291 | -0.310 |
|  |  | 2 | -0.312 | -0.281 | -0.223 | -0.272 | -0.279 |
|  |  | 3 | -0.246 | -0.197 | -0.201 | -0.215 | -0.187 |
|  | 0.0 to 1.0 | 1 | -2.393 | -3.133 | -3.931 | -3.152 | -2.616 |
|  |  | 2 | -2.230 | -1.775 | -2.992 | -2.332 | -1.369 |
|  |  | 3 | -1.937 | -1.706 | -2.958 | -2.200 | -1.358 |
|  | 1.0 to 12.0 | 1 | -0.132 | -0.033 | 0.073 | -0.031 | -0.101 |
|  |  | 2 | -0.137 | -0.145 | 0.029 | -0.084 | -0.180 |
|  |  | 3 | -0.092 | -0.060 | 0.050 | -0.034 | -0.081 |
| Sulfamethoxazole | 0 to 0.1 | 1 | -41.648 | -43.772 | -43.950 | -43.123 | -21.412 |
|  |  | 2 | -38.865 | -40.446 | -48.205 | -42.505 | 12.236 |
|  |  | 3 | -48.971 | -40.017 | -47.225 | -45.405 | 11.049 |
|  | 0.1 to 1.0 | 1 | -0.897 | -0.433 | -0.027 | -0.452 | 0.640 |
|  |  | 2 | -0.247 | -0.312 | 0.620 | 0.020 | -1.195 |
|  |  | 3 | 0.756 | -0.457 | 0.555 | 0.285 | -0.574 |
|  | 1.0 to 6.0 | 1 | -0.077 | -0.393 | -0.334 | -0.268 | -0.157 |
|  |  | 2 | -0.154 | -0.418 | -0.594 | -0.389 | 0.257 |
|  |  | 3 | -0.114 | -0.400 | -0.274 | -0.262 | -0.067 |
|  | 6.0 to 12.0 | 1 | -0.077 | -0.519 | -0.551 | -0.445 | 0.187 |
|  |  | 2 | -0.154 | -0.573 | -0.358 | -0.424 | -0.299 |
|  |  | 3 | -0.114 | -0.573 | -0.632 | -0.518 | 0.041 |
|  | 0.0 to 12.0 | 1 | -0.579 | -0.821 | -0.783 | -0.728 | -0.102 |
|  |  | 2 | -0.577 | -0.821 | -0.782 | -0.727 | -0.030 |
|  |  | 3 | -0.573 | -0.821 | -0.782 | -0.725 | 0.041 |
|  | 0.0 to 1.0 | 1 | -4.972 | -4.767 | -4.419 | -4.719 | -1.565 |
|  |  | 2 | -4.109 | -4.325 | -4.262 | -4.232 | 0.148 |
|  |  | 3 | -4.217 | -4.413 | -4.223 | -4.284 | 0.588 |
|  | 1.0 to 12.0 | 1 | -0.179 | -0.462 | -0.452 | -0.365 | 0.031 |
|  |  | 2 | -0.256 | -0.502 | -0.465 | -0.408 | -0.047 |
|  |  | 3 | -0.242 | -0.494 | -0.469 | -0.402 | -0.008 |

**Table S3.** Antibiotic kinetic degradation significance values. Subscripts on conditions denote the time point(s) or rates of change being compared in days. The following (*) signify the following levels of significance: p-values < 0.05 (*), < 0.01 (**), < 0.001 (***).

| Variable | Condition | Ciprofloxacin *p*-value | | Sulfamethoxazole *p*-value | |
| --- | --- | --- | --- | --- | --- |
|  |  | Shapiro-Wilk  (Normality) | Significance | Shapiro-Wilk  (Normality) | Significance |
| Independent | [ABC]_0.1_ vs. [X]_0.1_ | 0.610 | 0.283 | 0.185 | **0.016*** |
|  | [ABC]_1.0_ vs. [X]_1.0_ | **0.031*** | 0.400 | 0.587 | **0.004**** |
|  | [ABC]_6.0_ vs. [X]_6.0_ | 0.674 | 0.305 | 0.573 | **0.007**** |
|  | [ABC]_12.0_ vs. [X]_12.0_ | 0.395 | 0.994 | 0.126 | **<0.0001***** |
|  | [ABC]_0.0-0.1_ vs. [X]_0.0-0.1_ | 0.609 | 0.284 | 0.184 | **0.016*** |
|  | [ABC]_0.1-1.0_ vs. [X]_0.1-1.0_ | 0.144 | 0.160 | 0.970 | 0.603 |
|  | [ABC]_1.0-6.0_ vs. [X]_1.0-6.0_ | 0.644 | 0.605 | 0.556 | 0.075 |
|  | [ABC]_6.0-12.0_ vs. [X]_6.0-12.0_ | 0.821 | 0.109 | 0.641 | **0.040*** |
|  | [ABC]_0.0-12.0_ vs. [X]_0.0-12.0_ | 0.394 | 0.988 | 0.137 | **<0.0001***** |
|  | [ABC]_0.0-1.0_ vs. [X]_0.0-1.0_ | **0.031*** | 0.400 | 0.586 | **0.004**** |
|  | [ABC]_1.0-12.0_ vs. [X]_1.0-12.0_ | 0.197 | 0.111 | 0.864 | **<0.0005***** |
| Dependent | [ABC]_0.0_ vs. [ABC]_0.1_ | 0.605 | **0.010**** | 0.385 | **<0.0005***** |
|  | [ABC]_0.1_ vs. [ABC]_1.0_ | 0.076 | **0.019*** | 0.689 | 0.842 |
|  | [ABC]_1.0_ vs. [ABC]_6.0_ | 0.133 | **0.010**** | 0.075 | **0.018*** |
|  | [ABC]_6.0_ vs. [ABC]_12.0_ | 0.968 | 0.265 | 0.408 | **0.004**** |
|  | [ABC]_0.0_ to [ABC]_12.0_ | 0.372 | **<0.0001***** | **<0.0005***** | **<0.000005***** |
|  | [X]_0.0_ vs. [X]_0.1_ | 0.509 | 0.056 | 0.060 | 0.962 |
|  | [X]_0.1_ vs. [X]_1.0_ | 0.698 | 0.055 | 0.650 | 0.565 |
|  | [X]_1.0_ vs. [X]_6.0_ | 0.959 | 0.259 | 0.398 | 0.944 |
|  | [X]_6.0_ vs. [X]_12.0_ | 0.496 | 0.249 | 0.569 | 0.889 |
|  | [X]_0.0_ to [X]_12.0_ | 0.276 | **<0.001***** | 0.202 | 0.969 |

**Table S4.** Base-10 log transformed biofilm TCC data. Sample column represents the specific order that samples were taken in. Raw values prior to transformation can be found in Table S1.

| Associated antibiotic | Time (days) | Sample ID | log_10_[A] | log_10_[B] | log_10_[C] | log_10_[ABC] | log_10_[D] |
| --- | --- | --- | --- | --- | --- | --- | --- |
| Ciprofloxacin | 0.0 | 1 | 5.806 | 3.782 | 3.998 | 5.340 | 4.507 |
|  |  | 2 | 5.937 | 4.039 | 4.092 | 5.471 | 4.339 |
|  |  | 3 | 6.017 | 4.059 | 4.163 | 5.550 | 4.368 |
|  | 1.0 | 1 | 5.716 | 4.268 | 4.700 | 5.293 | 4.285 |
|  |  | 2 | 5.798 | 3.819 | 4.243 | 5.337 | 4.664 |
|  |  | 3 | 5.923 | 3.978 | 5.161 | 5.520 | 4.591 |
|  | 12.0 | 1 | 6.044 | 4.392 | 5.706 | 5.738 | 4.347 |
|  |  | 2 | 6.492 | 4.308 | 5.970 | 6.131 | 4.615 |
|  |  | 3 | 6.525 | 4.519 | 5.745 | 6.118 | 4.502 |
| Sulfamethoxazole | 0.0 | 1 | 5.026 | 4.544 | 5.099 | 4.949 | 4.507 |
|  |  | 2 | 4.922 | 4.803 | 5.679 | 5.319 | 4.339 |
|  |  | 3 | 4.562 | 5.663 | 5.459 | 5.418 | 4.368 |
|  | 1.0 | 1 | 5.292 | 4.967 | 5.359 | 5.236 | 4.285 |
|  |  | 2 | 5.011 | 4.669 | 5.533 | 5.213 | 4.664 |
|  |  | 3 | 5.121 | 4.377 | 5.268 | 5.056 | 4.591 |
|  | 12.0 | 1 | 3.999 | 4.045 | 5.287 | 4.854 | 4.347 |
|  |  | 2 | 4.104 | 4.417 | 5.147 | 4.776 | 4.615 |
|  |  | 3 | 5.345 | 4.254 | 5.192 | 5.120 | 4.502 |

**Table S5.** Calculated rates of change in biofilm TCCs. Rates of change were determined by calculating the slope after the base-10 logged TCC data. Δt represents change in time in days. Sample ID column represents the specific order that samples were taken in, corresponding with Table S4.

| Associated antibiotic | Δt (days) | Sample ID | Δlog_10_[A] | Δlog_10_[B] | Δlog_10_[C] | Δlog_10_[ABC] | Δlog_10_[D] |
| --- | --- | --- | --- | --- | --- | --- | --- |
| Ciprofloxacin | 0.0 to 1.0 | 1 | -0.090 | 0.486 | 0.702 | -0.046 | -0.222 |
|  |  | 2 | -0.139 | -0.220 | 0.152 | -0.134 | 0.325 |
|  |  | 3 | -0.093 | -0.081 | 0.997 | -0.030 | 0.224 |
|  | 1.0 to 12.0 | 1 | 0.030 | 0.011 | 0.091 | 0.040 | 0.006 |
|  |  | 2 | 0.063 | 0.044 | 0.157 | 0.072 | -0.005 |
|  |  | 3 | 0.055 | 0.049 | 0.053 | 0.054 | -0.008 |
|  | 0.0 to 12.0 | 1 | 0.020 | 0.051 | 0.142 | 0.033 | -0.013 |
|  |  | 2 | 0.046 | 0.022 | 0.156 | 0.055 | 0.023 |
|  |  | 3 | 0.042 | 0.038 | 0.132 | 0.047 | 0.011 |
| Sulfamethoxazole | 0.0 to 1.0 | 1 | 0.266 | 0.423 | 0.259 | 0.287 | -0.222 |
|  |  | 2 | 0.090 | -0.133 | -0.147 | -0.105 | 0.325 |
|  |  | 3 | 0.559 | -1.286 | -0.192 | -0.362 | 0.224 |
|  | 1.0 to 12.0 | 1 | -0.117 | -0.084 | -0.007 | -0.035 | 0.006 |
|  |  | 2 | -0.082 | -0.023 | -0.035 | -0.040 | -0.005 |
|  |  | 3 | 0.020 | -0.011 | -0.007 | 0.006 | -0.008 |
|  | 0.0 to 12.0 | 1 | -0.086 | -0.042 | 0.016 | -0.008 | -0.013 |
|  |  | 2 | -0.068 | -0.032 | -0.044 | -0.045 | 0.023 |
|  |  | 3 | 0.065 | -0.117 | -0.022 | -0.025 | 0.011 |

**Table S6.** Biofilm TCC significance values. Subscripts on conditions denote the time point(s) or rates of change being compared. The following (*) signify the following levels of significance: p-values < 0.05 (*), < 0.01 (**), < 0.001 (***).

| Variable | Condition | Ciprofloxacin *p*-value | | Sulfamethoxazole *p*-value | |
| --- | --- | --- | --- | --- | --- |
|  |  | Shapiro-Wilk  (Normality) | Significance | Shapiro-Wilk  (Normality) | Significance |
| Independent | [ABC]_0.0-1.0_ vs. [D]_0.0-1.0_ | 0.549 | 0.355 | 0.547 | 0.540 |
|  | [ABC]_1.0-12.0_ vs. [D]_1.0-12.0_ | 0.910 | **0.005**** | 0.463 | 0.245 |
|  | [ABC]_0.0-12.0_ vs. [D]_0.0-12.0_ | 0.707 | **0.037*** | 0.258 | 0.093 |
| Dependent | [ABC]_0.0_ vs. [ABC]_1.0_ | 0.324 | 0.161 | 0.770 | 0.781 |
|  | [ABC]_1.0_ vs. [ABC]_12.0_ | 0.276 | **0.026*** | 0.194 | 0.253 |
|  | [ABC]_0.0_ to [ABC]_12.0_ | 0.064 | **0.002**** | 0.966 | 0.236 |
|  | [D]_0.0_ vs. [D]_1.0_ | 0.334 | 0.584 | 0.334 | 0.584 |
|  | [D]_1.0_ vs. [D]_12.0_ | 0.491 | 0.628 | 0.491 | 0.628 |
|  | [D]_0.0_ to [D]_12.0_ | 0.394 | 0.686 | 0.394 | 0.686 |

**Table S7.** Raw data for the genus level community composition for the ciprofloxacin experiment. The relative abundance found in Table 4 was generated by taking the relative abundance for each day, denoted by the subscript, where A, B, C, and D (control), are individual BWDRs, and then combined to create an average for each individual BWDR. ABC is not shown as it was generated by combining the relative abundances of A, B, and C and taking their average.

| Genus | [A_Day 0_] | [A_Day 1_] | [A_Day 12_] | [B_Day 0_] | [B_Day 1_] | [B_Day 12_] | [C_Day 0_] | [C_Day 1_] | [C_Day 12_] | [D_Day 0_] | [D_Day 1_] | [D_Day 12_] |
| --- | --- | --- | --- | --- | --- | --- | --- | --- | --- | --- | --- | --- |
| *[Eubacterium] fissicatena group* | 157 | 133 | 49 | 58 | 68 | 75 | 8 | 461 | 353 | 316 | 21 | 12 |
| *Acidovorax* | 10346 | 12444 | 8868 | 7948 | 10329 | 14041 | 2994 | 6157 | 18397 | 25278 | 18281 | 3559 |
| *Acinetobacter* | 16 | 10 | 2 | 0 | 0 | 0 | 13 | 0 | 0 | 0 | 8 | 4 |
| *Allorhizobium-Neorhizobium-Pararhizobium-Rhizobium* | 5953 | 5158 | 17528 | 15571 | 17105 | 2610 | 16525 | 6604 | 11925 | 2617 | 21128 | 2169 |
| *Aminobacter* | 34 | 24 | 40 | 16 | 13 | 52 | 11 | 0 | 14 | 0 | 8 | 33 |
| *Anaerocolumna* | 18 | 16 | 16 | 169 | 206 | 0 | 4 | 56 | 15 | 56 | 6 | 0 |
| *Anaerospora* | 121 | 100 | 0 | 0 | 5 | 0 | 0 | 27 | 32 | 0 | 0 | 0 |
| *Anaerovorax* | 849 | 980 | 202 | 76 | 289 | 176 | 29 | 942 | 711 | 461 | 63 | 15 |
| *Ancylobacter* | 6 | 8 | 0 | 0 | 0 | 6 | 0 | 0 | 0 | 0 | 0 | 0 |
| *Aquabacterium* | 85 | 93 | 337 | 533 | 544 | 28 | 231 | 138 | 104 | 71 | 251 | 53 |
| *Arenimonas* | 0 | 0 | 0 | 0 | 0 | 0 | 0 | 0 | 0 | 0 | 0 | 0 |
| *Asinibacterium* | 2898 | 997 | 504 | 149 | 148 | 365 | 469 | 17 | 56 | 10 | 507 | 569 |
| *Azospira* | 1524 | 1120 | 110 | 282 | 533 | 141 | 280 | 3254 | 4100 | 1617 | 329 | 49 |
| *Azospirillum* | 191 | 155 | 226 | 138 | 177 | 133 | 64 | 78 | 138 | 15 | 77 | 5 |
| *Bacillus* | 0 | 0 | 0 | 8 | 4 | 0 | 3 | 0 | 0 | 0 | 0 | 0 |
| *Blastomonas* | 55 | 45 | 0 | 0 | 0 | 15 | 0 | 12 | 0 | 0 | 0 | 0 |
| *Bordetella* | 0 | 0 | 0 | 0 | 0 | 0 | 0 | 0 | 0 | 0 | 0 | 0 |
| *Bosea* | 0 | 0 | 5 | 10 | 13 | 0 | 0 | 48 | 97 | 12 | 0 | 0 |
| *Bradyrhizobium* | 0 | 0 | 0 | 0 | 0 | 0 | 3 | 0 | 0 | 0 | 0 | 0 |
| *Brevundimonas* | 3769 | 2908 | 392 | 238 | 279 | 1973 | 429 | 49 | 54 | 14 | 714 | 1505 |
| *Bryobacter* | 20 | 2 | 61 | 0 | 45 | 3 | 0 | 0 | 17 | 0 | 0 | 3 |
| *Caedibacter* | 103 | 90 | 169 | 19 | 17 | 97 | 113 | 0 | 20 | 7 | 123 | 220 |
| *Candidatus Berkiella* | 0 | 0 | 0 | 0 | 0 | 0 | 0 | 0 | 0 | 0 | 0 | 0 |
| *Candidatus Methylospira* | 4 | 0 | 4 | 0 | 0 | 0 | 0 | 0 | 0 | 0 | 0 | 0 |
| *Candidatus Obscuribacter* | 498 | 1254 | 124 | 3249 | 598 | 549 | 26 | 868 | 5 | 2010 | 11 | 0 |
| *Candidatus Paracaedibacter* | 615 | 622 | 1541 | 122 | 139 | 537 | 820 | 32 | 64 | 12 | 1039 | 1115 |
| *Caulobacter* | 2316 | 2711 | 715 | 1018 | 1203 | 2045 | 1087 | 1750 | 2404 | 908 | 2054 | 691 |
| *Cellulomonas* | 381 | 320 | 462 | 169 | 218 | 621 | 7 | 128 | 185 | 38 | 16 | 34 |
| *Chryseobacterium* | 449 | 421 | 1173 | 228 | 303 | 70 | 530 | 156 | 157 | 111 | 908 | 235 |
| *Clostridium sensu stricto 13* | 0 | 0 | 0 | 0 | 2 | 0 | 0 | 0 | 0 | 0 | 0 | 0 |
| *Corynebacterium* | 0 | 0 | 0 | 2 | 0 | 0 | 0 | 0 | 0 | 0 | 0 | 0 |
| *Cupriavidus* | 263 | 200 | 74 | 26 | 32 | 179 | 529 | 240 | 318 | 82 | 627 | 490 |
| *Curvibacter* | 349 | 302 | 0 | 0 | 0 | 127 | 0 | 0 | 0 | 0 | 0 | 0 |
| *Dechloromonas* | 2420 | 3543 | 4281 | 3201 | 4064 | 819 | 1461 | 62066 | 34225 | 47097 | 2032 | 213 |
| *Delftia* | 535 | 579 | 2596 | 4797 | 5322 | 33 | 2323 | 4596 | 2274 | 2194 | 3707 | 142 |
| *Desulfosporosinus* | 66 | 47 | 0 | 0 | 10 | 9 | 0 | 15 | 15 | 22 | 2 | 0 |
| *Desulfovibrio* | 0 | 0 | 0 | 0 | 0 | 0 | 0 | 0 | 0 | 0 | 0 | 0 |
| *Devosia* | 1551 | 1549 | 1220 | 286 | 409 | 1663 | 560 | 221 | 612 | 48 | 830 | 1475 |
| *DSSD61* | 42 | 32 | 0 | 0 | 0 | 31 | 0 | 0 | 0 | 0 | 0 | 14 |
| *Edaphobaculum* | 86 | 64 | 5 | 0 | 0 | 182 | 28 | 0 | 18 | 0 | 55 | 121 |
| *Ensifer* | 0 | 0 | 0 | 0 | 0 | 0 | 0 | 0 | 0 | 0 | 0 | 0 |
| *Enterococcus* | 0 | 0 | 0 | 0 | 0 | 0 | 0 | 0 | 0 | 0 | 0 | 0 |
| *Ferrovibrio* | 33 | 27 | 31 | 14 | 8 | 21 | 7 | 0 | 0 | 0 | 6 | 82 |
| *Fibrisoma* | 4194 | 3017 | 852 | 383 | 367 | 2919 | 154 | 17 | 20 | 7 | 229 | 1708 |
| *Fonticella* | 990 | 1727 | 693 | 276 | 505 | 138 | 21 | 4445 | 1405 | 2578 | 59 | 8 |
| *Galbitalea* | 1722 | 1329 | 883 | 136 | 172 | 2111 | 270 | 4 | 30 | 5 | 306 | 1532 |
| *Gemmata* | 0 | 0 | 0 | 0 | 0 | 0 | 0 | 0 | 0 | 0 | 0 | 0 |
| *Geobacillus* | 0 | 0 | 0 | 0 | 0 | 0 | 0 | 0 | 0 | 0 | 0 | 0 |
| *Hephaestia* | 46 | 34 | 5 | 0 | 0 | 20 | 14 | 11 | 10 | 0 | 12 | 31 |
| *Herbinix* | 23 | 14 | 24 | 33 | 78 | 0 | 0 | 13 | 46 | 10 | 12 | 0 |
| *Herminiimonas* | 0 | 0 | 0 | 0 | 0 | 0 | 8 | 0 | 0 | 0 | 22 | 13 |
| *Hyphomicrobium* | 16 | 12 | 0 | 0 | 0 | 62 | 0 | 0 | 0 | 0 | 3 | 8 |
| *IMCC26207* | 38 | 22 | 0 | 0 | 0 | 109 | 0 | 0 | 0 | 0 | 0 | 0 |
| *JGI 0001001-H03* | 0 | 0 | 0 | 0 | 0 | 0 | 0 | 0 | 0 | 0 | 3 | 0 |
| *Klebsiella* | 2193 | 1462 | 25 | 718 | 660 | 45 | 544 | 3328 | 3566 | 2024 | 587 | 5 |
| *Lacibacter* | 24 | 16 | 0 | 0 | 0 | 19 | 0 | 0 | 0 | 0 | 0 | 0 |
| *Lactobacillus* | 0 | 0 | 0 | 0 | 0 | 0 | 0 | 0 | 0 | 0 | 0 | 0 |
| *Lacunisphaera* | 10 | 15 | 0 | 2 | 3 | 9 | 3 | 172 | 339 | 120 | 4 | 0 |
| *Legionella* | 20 | 11 | 0 | 0 | 0 | 15 | 0 | 8 | 15 | 5 | 0 | 6 |
| *Leptospira* | 3 | 5 | 3 | 0 | 0 | 6 | 2 | 0 | 0 | 0 | 6 | 14 |
| *Luteitalea* | 61 | 51 | 0 | 0 | 0 | 69 | 0 | 0 | 0 | 0 | 0 | 0 |
| *Mesorhizobium* | 132 | 143 | 568 | 132 | 165 | 98 | 149 | 28 | 41 | 11 | 342 | 48 |
| *Methylomicrobium* | 0 | 0 | 0 | 0 | 3 | 0 | 0 | 0 | 0 | 0 | 0 | 0 |
| *Methylotenera* | 85 | 134 | 90 | 72 | 62 | 66 | 47 | 14 | 28 | 13 | 70 | 245 |
| *Methyloversatilis* | 277 | 259 | 529 | 128 | 129 | 349 | 269 | 111 | 171 | 57 | 298 | 382 |
| *Methylovulum* | 0 | 2 | 0 | 0 | 0 | 0 | 0 | 0 | 0 | 0 | 0 | 0 |
| *Microbacterium* | 0 | 0 | 0 | 0 | 0 | 0 | 0 | 0 | 0 | 0 | 0 | 0 |
| *Mobilitalea* | 130 | 139 | 67 | 33 | 62 | 16 | 0 | 33 | 117 | 20 | 0 | 0 |
| *Mycobacterium* | 106 | 82 | 0 | 0 | 0 | 122 | 0 | 0 | 0 | 0 | 0 | 46 |
| *Neochlamydia* | 318 | 324 | 337 | 76 | 108 | 303 | 280 | 7 | 10 | 0 | 351 | 224 |
| *Niveispirillum* | 0 | 0 | 0 | 0 | 0 | 0 | 0 | 0 | 0 | 0 | 0 | 0 |
| *Nordella* | 12941 | 9564 | 5161 | 561 | 690 | 11492 | 990 | 212 | 569 | 29 | 1148 | 6809 |
| *Novosphingobium* | 64 | 38 | 198 | 32 | 53 | 48 | 84 | 56 | 62 | 16 | 149 | 176 |
| *Nubsella* | 28 | 23 | 1514 | 238 | 347 | 22 | 48 | 502 | 638 | 367 | 115 | 11 |
| *Ohtaekwangia* | 1225 | 993 | 3 | 10 | 5 | 340 | 371 | 0 | 0 | 0 | 87 | 7 |
| *P3OB-42* | 372 | 281 | 122 | 0 | 8 | 279 | 30 | 0 | 2 | 0 | 45 | 244 |
| *Pajaroellobacter* | 27 | 36 | 0 | 0 | 0 | 215 | 0 | 0 | 0 | 0 | 0 | 0 |
| *Paracoccus* | 0 | 0 | 0 | 0 | 0 | 0 | 0 | 0 | 0 | 0 | 0 | 0 |
| *Pelosinus* | 55 | 93 | 31 | 4 | 12 | 26 | 0 | 36 | 24 | 17 | 0 | 0 |
| *Peredibacter* | 0 | 0 | 0 | 0 | 0 | 0 | 0 | 0 | 0 | 0 | 0 | 0 |
| *Phenylobacterium* | 364 | 303 | 154 | 48 | 39 | 204 | 71 | 41 | 40 | 5 | 92 | 191 |
| *Pigmentiphaga* | 0 | 0 | 0 | 0 | 0 | 0 | 0 | 0 | 0 | 0 | 0 | 0 |
| *Prosthecobacter* | 11 | 7 | 0 | 0 | 0 | 36 | 0 | 0 | 0 | 0 | 0 | 0 |
| *Pseudomonas* | 6109 | 6694 | 3942 | 38215 | 29748 | 856 | 26624 | 1180 | 1598 | 603 | 11726 | 228 |
| *Pseudorhodoferax* | 0 | 0 | 0 | 0 | 0 | 0 | 0 | 0 | 0 | 0 | 0 | 0 |
| *Pseudoxanthomonas* | 10880 | 10735 | 11399 | 1009 | 1224 | 6638 | 744 | 49 | 136 | 13 | 2056 | 21259 |
| *Reyranella* | 946 | 787 | 733 | 126 | 138 | 1416 | 289 | 36 | 90 | 10 | 355 | 809 |
| *Rhodobacter* | 63 | 42 | 0 | 0 | 0 | 15 | 0 | 0 | 6 | 0 | 6 | 0 |
| *Rhodococcus* | 789 | 658 | 18 | 0 | 8 | 918 | 235 | 246 | 400 | 135 | 312 | 540 |
| *Rhodopseudomonas* | 0 | 0 | 0 | 0 | 0 | 0 | 0 | 0 | 0 | 0 | 0 | 0 |
| *Roseomonas* | 118 | 85 | 29 | 4 | 5 | 84 | 8 | 5 | 13 | 0 | 8 | 128 |
| *Sedimentibacter* | 902 | 947 | 359 | 141 | 267 | 120 | 16 | 780 | 1490 | 780 | 42 | 9 |
| *Sediminibacterium* | 410 | 320 | 30 | 17 | 11 | 183 | 0 | 0 | 0 | 0 | 0 | 0 |
| *Shinella* | 0 | 0 | 0 | 0 | 0 | 0 | 0 | 0 | 0 | 0 | 0 | 0 |
| *SM1A02* | 0 | 0 | 7 | 0 | 0 | 0 | 0 | 0 | 5 | 0 | 4 | 32 |
| *Sphingobacterium* | 5 | 8 | 585 | 395 | 331 | 5 | 188 | 103 | 32 | 73 | 201 | 38 |
| *Sphingobium* | 329 | 290 | 1007 | 747 | 852 | 203 | 294 | 198 | 264 | 176 | 520 | 157 |
| *Sphingomonas* | 0 | 0 | 0 | 0 | 0 | 0 | 0 | 0 | 0 | 0 | 0 | 0 |
| *Sphingopyxis* | 1175 | 862 | 1872 | 307 | 432 | 699 | 250 | 163 | 246 | 27 | 316 | 866 |
| *Sphingorhabdus* | 1622 | 1202 | 25 | 9 | 4 | 997 | 24 | 15 | 24 | 0 | 36 | 200 |
| *Stenotrophomonas* | 293 | 218 | 1067 | 408 | 455 | 166 | 331 | 789 | 744 | 377 | 479 | 102 |
| *Streptomyces* | 0 | 0 | 0 | 0 | 0 | 0 | 0 | 7 | 0 | 0 | 0 | 0 |
| *Subgroup 10* | 0 | 0 | 0 | 0 | 0 | 0 | 0 | 0 | 0 | 0 | 0 | 0 |
| *Variovorax* | 141 | 98 | 37 | 24 | 0 | 59 | 25 | 0 | 0 | 0 | 54 | 0 |
| *Xanthobacter* | 0 | 0 | 4 | 0 | 0 | 5 | 0 | 0 | 0 | 0 | 0 | 0 |
| *Unassiged* | 17811 | 13986 | 8404 | 4375 | 4704 | 15340 | 5880 | 2031 | 3735 | 1359 | 6524 | 18134 |

**Table S8.** Raw data for the genus level community composition for the sulfamethoxazole experiment. The relative abundance found in Table 4 was generated by taking the relative abundance for each day, denoted by the subscript, where A, B, C, and D (control), are individual BWDRs, and then combined to create an average for each individual BWDR. ABC is not shown as it was generated by combining the relative abundances of A, B, and C and taking their average.

| Genus | [A_Day 0_] | [A_Day 1_] | [A_Day 12_] | [B_Day 0_] | [B_Day 1_] | [B_Day 12_] | [C_Day 0_] | [C_Day 1_] | [C_Day 12_] | [D_Day 0_] | [D_Day 1_] | [D_Day 12_] |
| --- | --- | --- | --- | --- | --- | --- | --- | --- | --- | --- | --- | --- |
| *[Eubacterium] fissicatena group* | 0 | 0 | 0 | 40 | 54 | 5 | 6 | 12 | 14 | 12 | 30 | 5 |
| *Acidovorax* | 1982 | 1855 | 1425 | 2850 | 3079 | 2343 | 5682 | 4897 | 3267 | 1175 | 3081 | 641 |
| *Acinetobacter* | 0 | 4 | 0 | 0 | 0 | 0 | 0 | 0 | 4 | 0 | 0 | 0 |
| *Allorhizobium-Neorhizobium-Pararhizobium-Rhizobium* | 22681 | 19697 | 9000 | 12910 | 14447 | 4357 | 19564 | 15066 | 1735 | 4477 | 12003 | 2605 |
| *Aminobacter* | 0 | 0 | 0 | 0 | 0 | 0 | 0 | 0 | 0 | 0 | 0 | 0 |
| *Anaerocolumna* | 0 | 7 | 0 | 118 | 159 | 41 | 43 | 38 | 9 | 41 | 29 | 0 |
| *Anaerospora* | 29 | 24 | 0 | 336 | 451 | 42 | 16 | 0 | 0 | 93 | 212 | 21 |
| *Anaerovorax* | 18 | 14 | 19 | 988 | 1230 | 134 | 106 | 58 | 28 | 242 | 371 | 4 |
| *Ancylobacter* | 0 | 0 | 7 | 0 | 8 | 0 | 0 | 0 | 7 | 0 | 0 | 0 |
| *Aquabacterium* | 0 | 0 | 0 | 0 | 0 | 0 | 0 | 0 | 37 | 0 | 0 | 0 |
| *Asinibacterium* | 804 | 800 | 1652 | 98 | 158 | 61 | 0 | 0 | 836 | 139 | 408 | 113 |
| *Azospira* | 30 | 40 | 15 | 930 | 1275 | 719 | 93 | 135 | 546 | 807 | 892 | 97 |
| *Azospirillum* | 0 | 0 | 5 | 15 | 18 | 6 | 61 | 39 | 5 | 0 | 3 | 0 |
| *Bordetella* | 0 | 0 | 0 | 0 | 0 | 0 | 0 | 0 | 33 | 0 | 0 | 0 |
| *Brevundimonas* | 71 | 85 | 84 | 411 | 523 | 206 | 122 | 107 | 177 | 79 | 126 | 39 |
| *Bryobacter* | 0 | 0 | 0 | 0 | 0 | 0 | 0 | 0 | 2 | 0 | 0 | 0 |
| *Caedibacter* | 14 | 8 | 13 | 0 | 4 | 0 | 13 | 10 | 40 | 26 | 44 | 9 |
| *Candidatus Obscuribacter* | 115 | 86 | 367 | 26 | 27 | 28 | 0 | 0 | 50 | 0 | 0 | 0 |
| *Candidatus Paracaedibacter* | 406 | 306 | 849 | 57 | 62 | 35 | 199 | 105 | 344 | 74 | 189 | 36 |
| *Caulobacter* | 937 | 1304 | 523 | 6000 | 6383 | 2821 | 1589 | 1240 | 1231 | 4202 | 3094 | 460 |
| *Cellulomonas* | 6 | 5 | 29 | 35 | 62 | 11 | 40 | 39 | 0 | 12 | 12 | 0 |
| *Chryseobacterium* | 362 | 569 | 39 | 314 | 455 | 81 | 457 | 182 | 501 | 162 | 580 | 50 |
| *Cupriavidus* | 15 | 18 | 17 | 935 | 928 | 527 | 2492 | 1841 | 161 | 1880 | 4290 | 1386 |
| *Dechloromonas* | 42 | 111 | 46 | 907 | 1710 | 1271 | 4374 | 2262 | 1006 | 1733 | 4895 | 1309 |
| *Delftia* | 25466 | 21915 | 2098 | 12935 | 15968 | 8463 | 8098 | 6538 | 679 | 5148 | 11841 | 2014 |
| *Desulfovibrio* | 284 | 305 | 156 | 281 | 491 | 319 | 614 | 479 | 87 | 57 | 169 | 86 |
| *Devosia* | 0 | 0 | 0 | 0 | 0 | 0 | 0 | 0 | 14 | 0 | 0 | 0 |
| *Edaphobaculum* | 76 | 122 | 153 | 50 | 64 | 106 | 8 | 8 | 165 | 59 | 106 | 19 |
| *Ensifer* | 0 | 0 | 0 | 0 | 0 | 0 | 264 | 327 | 425 | 0 | 0 | 0 |
| *Ferrovibrio* | 12 | 15 | 23 | 3 | 0 | 0 | 0 | 0 | 0 | 0 | 3 | 0 |
| *Fibrisoma* | 119 | 114 | 373 | 0 | 3 | 0 | 0 | 0 | 122 | 0 | 0 | 0 |
| *Fonticella* | 163 | 270 | 118 | 262 | 294 | 25 | 31 | 13 | 38 | 396 | 559 | 11 |
| *Galbitalea* | 0 | 0 | 32 | 0 | 0 | 0 | 0 | 0 | 52 | 0 | 0 | 0 |
| *Gemmata* | 0 | 3 | 41 | 0 | 0 | 0 | 0 | 0 | 4 | 0 | 0 | 0 |
| *Hephaestia* | 0 | 0 | 0 | 11 | 14 | 0 | 0 | 0 | 0 | 0 | 0 | 0 |
| *Herbinix* | 0 | 21 | 0 | 223 | 308 | 44 | 97 | 95 | 46 | 98 | 114 | 12 |
| *Herminiimonas* | 7 | 6 | 0 | 0 | 0 | 0 | 0 | 0 | 0 | 0 | 0 | 0 |
| *Klebsiella* | 1699 | 1979 | 377 | 1582 | 1827 | 551 | 1241 | 469 | 50 | 1310 | 3410 | 1417 |
| *Lacibacter* | 0 | 0 | 0 | 0 | 0 | 0 | 0 | 0 | 5 | 0 | 0 | 0 |
| *Lacunisphaera* | 0 | 0 | 0 | 3 | 4 | 0 | 0 | 0 | 6 | 27 | 7 | 2 |
| *Mesorhizobium* | 71 | 53 | 69 | 44 | 37 | 8 | 17 | 15 | 95 | 9 | 22 | 0 |
| *Methylotenera* | 39 | 56 | 16 | 161 | 138 | 78 | 54 | 37 | 167 | 47 | 43 | 7 |
| *Methyloversatilis* | 15 | 16 | 46 | 15 | 18 | 14 | 13 | 0 | 0 | 0 | 0 | 0 |
| *Mobilitalea* | 12 | 0 | 19 | 94 | 164 | 27 | 32 | 0 | 0 | 50 | 45 | 8 |
| *Neochlamydia* | 67 | 57 | 59 | 0 | 0 | 0 | 0 | 0 | 321 | 0 | 0 | 0 |
| *Nordella* | 59 | 31 | 234 | 0 | 0 | 5 | 0 | 0 | 120 | 0 | 0 | 0 |
| *Novosphingobium* | 29 | 27 | 42 | 52 | 56 | 25 | 36 | 31 | 28 | 27 | 80 | 8 |
| *Nubsella* | 281 | 291 | 111 | 303 | 333 | 188 | 147 | 65 | 573 | 120 | 323 | 73 |
| *Ohtaekwangia* | 47 | 84 | 174 | 213 | 326 | 160 | 0 | 0 | 12 | 0 | 3 | 0 |
| *P3OB-42* | 0 | 0 | 14 | 0 | 0 | 0 | 0 | 0 | 3 | 0 | 0 | 0 |
| *Pajaroellobacter* | 0 | 0 | 0 | 0 | 0 | 0 | 0 | 0 | 27 | 0 | 0 | 0 |
| *Paracoccus* | 0 | 0 | 0 | 5 | 0 | 0 | 0 | 0 | 0 | 0 | 0 | 0 |
| *Pelosinus* | 13 | 14 | 0 | 98 | 117 | 19 | 73 | 37 | 8 | 66 | 212 | 16 |
| *Peredibacter* | 0 | 0 | 0 | 0 | 2 | 0 | 0 | 0 | 0 | 0 | 0 | 0 |
| *Phenylobacterium* | 7 | 0 | 14 | 15 | 7 | 5 | 0 | 12 | 33 | 0 | 11 | 0 |
| *Pigmentiphaga* | 0 | 0 | 0 | 17 | 10 | 7 | 0 | 0 | 0 | 0 | 0 | 0 |
| *Pseudomonas* | 968 | 2086 | 833 | 1589 | 1525 | 922 | 1609 | 1275 | 4162 | 536 | 845 | 301 |
| *Pseudorhodoferax* | 8 | 0 | 59 | 0 | 6 | 0 | 0 | 0 | 144 | 0 | 0 | 0 |
| *Pseudoxanthomonas* | 0 | 0 | 23 | 27 | 41 | 15 | 11 | 17 | 127 | 0 | 11 | 4 |
| *Reyranella* | 146 | 149 | 400 | 5 | 7 | 0 | 13 | 9 | 124 | 0 | 0 | 0 |
| *Rhodococcus* | 0 | 2 | 4 | 50 | 57 | 16 | 0 | 0 | 4 | 0 | 0 | 0 |
| *Sedimentibacter* | 27 | 58 | 9 | 2992 | 4016 | 608 | 75 | 81 | 47 | 673 | 1364 | 92 |
| *Sediminibacterium* | 0 | 0 | 0 | 0 | 0 | 0 | 0 | 0 | 6 | 0 | 0 | 0 |
| *Shinella* | 0 | 0 | 0 | 61 | 82 | 55 | 0 | 0 | 0 | 45 | 90 | 19 |
| *Sphingobacterium* | 0 | 0 | 7 | 41 | 36 | 10 | 0 | 0 | 40 | 17 | 37 | 9 |
| *Sphingobium* | 239 | 233 | 385 | 311 | 281 | 116 | 56 | 41 | 956 | 23 | 37 | 7 |
| *Sphingomonas* | 0 | 0 | 0 | 0 | 0 | 0 | 0 | 0 | 55 | 0 | 0 | 0 |
| *Sphingopyxis* | 75 | 68 | 350 | 110 | 131 | 46 | 6 | 6 | 48 | 0 | 8 | 0 |
| *Sphingorhabdus* | 0 | 12 | 18 | 0 | 0 | 0 | 0 | 0 | 30 | 0 | 0 | 0 |
| *Stenotrophomonas* | 297 | 261 | 46 | 1902 | 1992 | 2458 | 4004 | 2973 | 332 | 299 | 938 | 200 |
| *Variovorax* | 66 | 62 | 36 | 109 | 121 | 68 | 78 | 95 | 57 | 0 | 0 | 0 |
| Unassigned | 1010 | 796 | 4052 | 1505 | 1796 | 1115 | 2976 | 2042 | 4769 | 1231 | 1925 | 335 |

**Text S1: Water quality parameter methods.**

During the entirety of the experimental period, the following water quality parameters were continuously measured: temperature and pH (Accumet XL60), turbidity (Hach 2100Q turbidimeter, USA), UV absorbance at 600 nm (Orion^TM^ AquaMate UV-VIS Spectrophotometer, ThermoScientific, USA), total organic carbon (TOC) and total nitrogen (TN) (Shimazdu TOC analyzer, Shimadzu, USA), free chlorine (DR300 Pocket Colorimeter, Hach, USA), adenosine triphosphate (ATP) (Quench-Gone Aqueous (QGA) test kit (LuminUltra Technologies, Canada), with the Lumitester C-110 luminometer (Kikkoman, Japan), and TCCs (Bacterial Counting Kit for flow cytometry, Invitrogen, USA) according to their respective manufacturers’ protocols.

**Text S2: TCC and 16S rRNA methods for biofilm collection, quantification, and identification.**

Individual fixative solutions were prepared with 100 μL of glutaraldehyde (50 wt. %, 340855, Millipore Sigma) and 9.9 mL of DI water in a sterile falcon tube and stored in the dark at 4°C for a maximum of 21 days. Each swab was vortexed at 3000 rpm (Fisherbrand™ Digital Vortex Mixer) for 30 seconds inside the falcon tube and then subjected to sonication at a low frequency of 40 kHz (Branson Ultrasonics™ CPX1800, Danbury, CT) for 1 minute, followed by an additional vortexing for 30 seconds. To quantify the bacteria that were dislodged from the swab, a TCC method using the Bacterial Counting Kit for flow cytometry (Invitrogen, Carlsbad, CA USA) was modified as follows. Polystyrene microspheres (beads) (6 μm, B7277, Invitrogen, Carlsbad, CA USA) were sonicated in a water bath for 5 minutes for resuspension. At the same time, new samples (specimens) were prepared in 5 mL round base polystyrene flow cytometry tubes. Each specimen consisted of 1 mL of swab contents (bacteria) and 1 μL of SYTO^TM^ BC bacteria stain for 5 minutes, as well as 10 μL of beads once the staining and sonication were complete. All specimens were triturated by pipette. Control specimens to create gating thresholds consisted of unstained bacteria only (no beads or stain), beads and stain only with filtered DI water (no bacteria), and bacteria and stain (no beads). Analysis was performed on a SH800 cell sorter (Sony, Japan) at an excitation wavelength of 488 nm. In accordance with the manufacturer’s protocol, the bacteria frame was divided by the microsphere frame to determine the number of bacteria per 1000 μL of solution for a total of 100,000 events acquired. The resulting data analysis was performed using FlowJo™ (BD Biosciences, NJ, USA).

For bacterial species identification, a larger quantity of biomass was required to perform 16S rRNA sequencing. Removable pipe segments (surface area 151.29 cm^2^) were extracted at every time point of 0, 1, and 12 days. Each pipe segment was rinsed in DI water via vertical submersion and swabbed with sterile swabs until complete removal of visible biofilm. Swabs for each pipe segment were placed in a sterile, 15 mL nuclease-free tube (FroggaBio, ON, Canada) containing 5 mL of autoclaved DI water. Each set of swabs was vortexed at 3000 rpm (Fisherbrand™ Digital Vortex Mixer) for 30 seconds inside their nuclease-free tube and then subjected to sonication at a low frequency of 40 kHz (Branson Ultrasonics™ CPX1800, Danbury, CT) for 1 minute, followed by an additional vortexing for 30 seconds. Swabs were then removed and discarded. The nuclease-free tubes containing the suspended biofilms were then centrifuged at 3800 rpm for 10 minutes to create a pellet and the 4 mL of supernatant was discarded. All nuclease-free tubes were stored at -80°C until DNA extraction. DNA was extracted with the Sox DNA Isolation Kit (Metagenom Bio Inc.) according to the supplier’s recommendation. 16S rRNA V4 regions were PCR amplified using forward primer and reverse primer (515FB:5’-GTGYCAGCMGCCGCGGTAA-3’ and 806RB:5’-GGACTACNVGGGTWTCTAAT-3’, Walters et al., 2016). Illumina sequencing adapters and barcodes were added to the 5’ ends of the primers and each indexed primer pair contained a 5’ random addition of up to six random nucleotides to improve heterogeneity of the sequencing run, improving read quality and yield. PCR was set up in duplicate (25 μL each): 2.5 μL of 10 × standard Taq reaction buffer, 0.5 μL 10 mM dNTP, 0.2 μL Taq DNA polymerase (New England Labs), 0.2 μL BSA (20 mg mL^-1^, NEB), 5.0 μL of 1 μM forward and reverse primers respectively, 9.1 μL water and 2.5 μL eDNA (1-10 ng μL^-1^). PCR was run as follows: 94°C for 5 min, 35 cycles of 94°C for 30 sec, 50°C for 30 sec and 68°C for 1 min, and finally 68°C for 5 min. PCR amplicons were pooled and resolved in 2% agarose gel. 16S rRNA amplicons were gel-purified and then quantified using a Qubit dsDNA quantification Assay kit (ThermoFisher). The 16S rRNA amplicons were diluted to 4 nM, denatured and sequenced using a MiSeq 500-cycle sequencing v2 kit (Illumina, Cat. No. MS-102-2003). Demultiplexed sequences were processed using cutadapt (primer removal, Martin, 2011) DADA2 v.1.22 (sample inference, Callahan et al., 2016). Reads were truncated at decreasing quality scores and assembled (typically 225 on read R1 and 200 on R2 relative to the original read start position). After quality filtering (chimera, organelles) an amplified sequence variant (ASV) abundance table was constructed. This ASV table records the number of sequence reads for each amplified sequence variant (“species”) for each sample. Taxonomy was assigned to representative sequences using the naive Bayesian classifier implemented in the dada2::assignTaxonomy() function from DADA2 (Callahan et al., 2016) trained against the SILVA reference database release 138 (Quast et al., 2012).

**References**

Callahan, B. J., McMurdie, P. J., Rosen, M. J., Han, A. W., Johnson, A. J. A., & Holmes, S. P. (2016). DADA2: High-resolution sample inference from Illumina amplicon data. *Nature Methods*, *13*(7), 581-583.

Quast, C., Pruesse, E., Yilmaz, P., Gerken, J., Schweer, T., Yarza, P., Peplies, J., & Glöckner, F. O. (2012). The SILVA ribosomal RNA gene database project: improved data processing and web-based tools. *Nucleic Acids Research*, *41*(D1), D590-D596.

Walters, W., Hyde, E. R., Berg-Lyons, D., Ackermann, G., Humphrey, G., Parada, A., Gilbert, J. A., Jansson, J. K., Caporaso, J. G., Fuhrman, J. A., Apprill, A., & Knight, R. (2016). Improved bacterial 16S rRNA gene (V4 and V4-5) and fungal internal transcribed spacer marker gene primers for microbial community surveys. *Msystems*, *1*(1), e00009-15.
